# Supplementary material for: Cohort profile: maternal antecedents of adiposity and studying the transgenerational role of hyperglycaemia and insulin (MAASTHI)
Source: BMJ Open. 2022 Sep 21;12(9):e063794. doi: 10.1136/bmjopen-2022-063794 (PMC9494597; doi:10.1136/bmjopen-2022-063794)
Supplement: Author's manuscript [file bmjopen-2022-063794.draft_revisions.pdf]

BMJ Open is committed to open peer review. As part of this commitment we make the peer review history of every article we publish publicly available.

When an article is published we post the peer reviewers' comments and the authors' responses online. We also post the versions of the paper that were used during peer review. These are the versions that the peer review comments apply to.

The versions of the paper that follow are the versions that were submitted during the peer review process. They are not the versions of record or the final published versions. They should not be cited or distributed as the published version of this manuscript.

BMJ Open is an open access journal and the full, final, typeset and author-corrected version of record of the manuscript is available on our site with no access controls, subscription charges or pay-per-view fees (http://bmjopen.bmj.com).

If you have any questions on BMJ Open's open peer review process please email info.bmjopen@bmj.com

BMJ Open

Cohort Profile: Maternal antecedents of adiposity and studying the transgenerational role of hyperglycemia and insulin (MAASTHI)

Journal:	*BMJ Open*
Manuscript ID	bmjopen-2022-063794
Article Type:	Cohort profile
Date Submitted by the Author:	19-Apr-2022
Complete List of Authors:	Lobo, Eunice; Public Health Foundation of India<br>Ana, Yamuna; Public Health Foundation of India<br>Deepa, R; Public Health Foundation of India<br>Shriyan, Prafulla; Public Health Foundation of India<br>Sindhu, ND; Public Health Foundation of India<br>Karthik, Maithili; Public Health Foundation of India<br>Kinra, Sanjay; London School of Hygiene & Tropical Medicine<br>Murthy, GVS; Public Health Foundation of India, IIPH Hyderabad.<br>Babu, Giridhara R; Public Health Foundation of India; Wellcome Trust DBT India Alliance
Keywords:	DIABETES & ENDOCRINOLOGY, PUBLIC HEALTH, Health policy < HEALTH SERVICES ADMINISTRATION & MANAGEMENT

SCHOLARONE™
Manuscripts

I, the Submitting Author has the right to grant and does grant on behalf of all authors of the Work (as defined in the below author licence), an exclusive licence and/or a non-exclusive licence for contributions from authors who are: i) UK Crown employees; ii) where BMJ has agreed a CC-BY licence shall apply, and/or iii) in accordance with the terms applicable for US Federal Government officers or employees acting as part of their official duties; on a worldwide, perpetual, irrevocable, royalty-free basis to BMJ Publishing Group Ltd ("BMJ") its licensees and where the relevant Journal is co-owned by BMJ to the co-owners of the Journal, to publish the Work in this journal and any other BMJ products and to exploit all rights, as set out in our licence.

The Submitting Author accepts and understands that any supply made under these terms is made by BMJ to the Submitting Author unless you are acting as an employee on behalf of your employer or a postgraduate student of an affiliated institution which is paying any applicable article publishing charge ("APC") for Open Access articles. Where the Submitting Author wishes to make the Work available on an Open Access basis (and intends to pay the relevant APC), the terms of reuse of such Open Access shall be governed by a Creative Commons licence – details of these licences and which Creative Commons licence will apply to this Work are set out in our licence referred to above.

Other than as permitted in any relevant BMJ Author's Self Archiving Policies, I confirm this Work has not been accepted for publication elsewhere, is not being considered for publication elsewhere and does not duplicate material already published. I confirm all authors consent to publication of this Work and authorise the granting of this licence.

For peer review only

Full Title: Cohort Profile: Maternal antecedents of adiposity and studying the transgenerational role of hyperglycemia and insulin (MAASTHI)

Short title: Cohort profile: MAASTHI

Authors: Eunice Lobo[1], Yamuna Ana[1], R Deepa,[1] Prafulla Shriyan,[1] ND Sindhu,[1] Maithili Karthik,[1] Sanjay Kinra,[2] GVS Murthy,[3,4] Giridhara R. Babu[1,5]

Affiliations:

1. Indian Institute of Public Health-Bengaluru, Public Health Foundation of India (PHFI), Bengaluru, India
2. London School of Hygiene & Tropical Medicine and University College London Hospital, London, United Kingdom
3. International Centre for Eye Health, Department of Clinical Research, London School of Hygiene & Tropical Medicine, London, United Kingdom
4. Indian Institute of Public Health-Hyderabad, Public Health Foundation of India (PHFI), Hyderabad, India
5. Wellcome Trust-DBT India Alliance Intermediate Research Fellow in Public Health, Hyderabad, India

* **Corresponding author**: Dr Giridhara R. Babu,

Address: Indian Institute of Public Health-Bengaluru, Public Health Foundation of India (PHFI), Besides Leprosy Hospital, 1st Cross, Magadi Road, Bengaluru, India - 560023

Email: epigiridhar@gmail.com

For peer review only

Abstract

Purpose: The Maternal Antecedents of Adiposity and Studying the transgenerational role of Hyperglycemia and Insulin (MAASTHI) cohort in Bengaluru, South India, aims to understand the transgenerational role of increased circulating glucose levels or hyperglycemia and other nutrients and psychosocial environment, on the risk of childhood obesity, as an early marker of chronic diseases.

Participants: Through this paper, we describe the baseline characteristics of the cohort participants and their children, along with future plans and challenges. A total of 5694 pregnant women were screened, with 4862 (85.4%) eligible pregnant women recruited at baseline. We assessed anthropometry, Haemoglobin status, Oral Glucose Tolerance Test (OGTT), dietary practices, depressive symptoms using the Edinburgh Postnatal Depression Scale (EPDS), and social support in all women. Follow-up visits involved assessing anthropometry and the health profile of mothers and children.

Findings to date: Among the 4862 eligible participants recruited, 3260 (67%) underwent OGTT, while 2962 participants completed OGTT (90.9%). During pregnancy, 9.7% of women were obese (>90th percentile of skinfold thickness), and 14.3% had Gestational Diabetes Mellitus (GDM). Moreover, 6.2% and 16.8% of women had symptoms suggestive of depression during pregnancy and the immediate post-natal period, respectively. We found that 3.3% of children were Small for Gestational Age (SGA), 10.8% were Large for Gestational Age (LGA), and 9.7% of children were obese at birth.

Future plans: We have completed recruitment and baseline data collection in 2019, and are conducting annual follow-up until age 4 of the participant's children. For delineating causal pathways of childhood obesity, blood aliquots are stored in the biorepository. The study will inform policy formulation and community awareness in the prevention and control of Non-Communicable Diseases (NCDs) and health promotion.

Keywords: *Gestational diabetes, obesity, birth cohort, psychosocial environment, low birth weight, India*

For peer review only

Strengths and limitations of this study

- MAASTHI is a well-established cohort study documenting the life course trajectory in the genesis of NCDs for important exposure contrasts experienced by women very early on in their pregnancy and continues to document the outcomes across the process of pregnancy, childbirth and early childhood, including assessing important milestones of the child.
- This is the largest prospective birth cohort in the urban public sector on maternal and child health in South India, with a large sample size over a relatively long period of follow-up.
- Blood aliquots are stored in the biorepository for future studies on childhood obesity.
- Operational issues in terms of incomplete OGTT and loss-to-follow-up after delivery are the limitations of conducting a study in public health facilities.
- Our cohort will inform policy formulation and community awareness in the prevention and control of Non-Communicable Diseases (NCDs) and health promotion.

For peer review only - http://bmjopen.bmj.com/site/about/guidelines.xhtml

Introduction to MAASTHI cohort

According to the recent global disease burden study, type 2 diabetes mellitus (T2DM) is the fifth commonest disease affecting Indians (1). T2DM is projected to affect 70 million Indians by the year 2025 (2-7) at a relatively younger age compared to high-income countries. The increasing prevalence of Gestational diabetes mellitus (GDM) directly impacts the T2DM burden (6). Findings from the Hyperglycemia and Adverse Pregnancy Outcome (HAPO) study initially confirmed the association between maternal glucose levels and neonatal adiposity (8-11). These results conform to the theoretical frameworks of 'fuel-mediated teratogenesis', 'thrifty phenotype', and thrifty genotype' hypotheses (2, 8, 11-15), which explain the trans-generational nature of obesity-hyperglycemia. Even the Parthenon Birth cohort and others (16-18) have provided initial pieces of evidence regarding this association from low-and-middle-income countries (LMICs). Thus, it becomes necessary to update the recent findings and, more importantly, capture the evidence from resource-constrained, high burden countries such as India. Given the rapid demographical and epidemiological transition in India, there is a pressing need to capture the evidence on the obesity-hyperglycemia burden (19).

In LMICs, children who have low birth weight (LBW) and early undernutrition are known to have rebound adiposity during early adolescence (20). Therefore, it is important to capture the life course trajectories of the underweight and obesity-led hyperglycemia epidemics in LMICs. The risk factors of LBW may also be modifiable to prevent ensuing obesity epidemics in these countries. Among these risk factors, undernutrition in pregnancy affects nearly 28% of newborns having LBW in South Asia (21, 22). Evidence from the high-income countries suggests a putative role for the psychosocial environment and weight of the newborn (23, 24).

Aligned with the existing evidence from the high-income countries, we piloted the feasibility of conducting a multi-centric cohort study in the urban public health facilities of Bengaluru. Informed from the results of the pilot study (25), the principal investigator (GRB) obtained funding to set up a large birth cohort

For peer review only - http://bmjopen.bmj.com/site/about/guidelines.xhtml

titled ‘Maternal antecedents of adiposity and studying the transgenerational role of hyperglycemia and insulin’ (MAASTHI). The primary objective of the cohort is to investigate the effect of glucose levels in pregnancy on skinfold thickness (adiposity) in infancy as a marker of future obesity and diabetes in offspring, while the secondary objective is to assess the association between the psychosocial environment of mothers and adverse neonatal outcomes including adiposity. By capturing evidence from the mothers and children of low-and-middle-income status visiting health facilities in the public sector, MAASTHI will provide a contextual evidence base resulting in obesity-hyperglycemia in India (9, 25).

For peer review only

For peer review only - http://bmjopen.bmj.com/site/about/guidelines.xhtml

Cohort description

Study design, sample size, study site and participants

We started the recruitment in the prospective cohort study from April 2016 in the urban public health facilities of Bengaluru, a metropolitan city with 13 million population in South India. The study protocol with the details of recruitment and methodology is published elsewhere (26). With the assumption of a 5% incidence of childhood obesity at birth in India, a relative risk of 1.5 in the hyperglycaemic group, our estimated sample size with 80% power required to detect a difference at a 95% confidence level was 2936 participants. Considering loss to follow-up by about 60%, we had aimed to recruit 5000 pregnant women. We started recruitment in a 300-bedded public health facility (Jayanagar General Hospital) in Bengaluru corporation. The cohort is established in 14 public health facilities in Bengaluru, Karnataka viz. Jayanagar General Hospital, Bagaluru referral Hospital, Srirampura referral Hospital, Anjanappa Garden UPHC, Banashankari Referral Hospital, Bapuji Nagar UPHC, D J Halli UPHC, KC General Hospital, Magadi road UPHC, Pantharapalaya UPHC, Robertson pet UPHC, Siddayya Referral Hospital, Sirsi Circle UPHC, and Tavrekeri UPHC.

All pregnant women between 14 to 36 weeks of gestation from the ages of 18 to 45 years visiting the public health facilities were approached to participate in the study. After obtaining written informed consent, we recruited the women only if they planned to deliver in the study health facility and were available for future follow-ups in Bengaluru. Exclusion criteria included a history of major co-existing diseases as HIV/ADIS, Hepatitis B, diabetes, and the inability to complete the oral glucose tolerance test (OGTT) within 24 to 36 weeks of gestation.

Patient and public involvement

No patient involvement.

For peer review only

For peer review only - http://bmjopen.bmj.com/site/about/guidelines.xhtml

Data collection

Recruitment and follow-up

We approached 5694 pregnant women and collected the baseline data from 4862 eligible pregnant women and 2873 women who delivered during the period of April 2016 to June 2019. A brief overview of study recruitment, retention and at birth follow up is presented in the fig. 1.

After obtaining written informed consent, eligible pregnant women were recruited into the study when they completed 14 weeks of gestational age, and face-to-face interviews were done by trained research assistants ensuring privacy and confidentiality.

What has been measured?

We have collected socio-demographic details, 24-hour dietary recall, dietary habits, use of tobacco and alcohol (own and spouse) and physical activity in the women. In addition to obtaining a family history of diabetes and other cardiovascular diseases (CVD), we obtained an obstetric history from all the pregnant women (Table 1). Additionally, we have assessed the psychosocial environment using validated versions of the Edinburgh Postnatal Depression Scale (EPDS) and social support scale (27). Between 24 to 36 weeks, all the women were invited to undergo OGTT. GDM was classified using the World Health Organisation (WHO) diagnostic criteria (28). We also measured blood pressure, assessed body mass index (BMI), and measured hemoglobin in all the women (Table 1).

The follow-up visits began soon after the delivery. We recorded the health status, morbidities, or any other illness of the baby, feeding and mother's follow up assessments. The measurements on skinfold thickness, circumferences, and weight, length in infants are taken at birth and 14 weeks, corresponding to the child's immunization visits to the public health facility. Further follow-up visits of the children

For peer review only - http://bmjopen.bmj.com/site/about/guidelines.xhtml

include anthropometric assessment annually at ages 1, 2, 3 and 4; along with other assessments mentioned in Table 1.

Table 1: Assessments and data collected among the participants of MAASTHI birth cohort, Bengaluru 2016

Time	***Variable***
Mother: *14 to 36 weeks*	Socio-demographic details<br>Family medical history<br>Physical activity<br>Tobacco/Alcohol use (Self and spouse)<br>Dietary habits/24-hour recall<br>Obstetrics history<br>Assessment of depressive symptoms (using EPDS) and social support<br>Medications/supplements taken<br>Blood pressure<br>Anthropometry measurements<br>• Weight<br>• Height<br>• Biceps skinfold thickness<br>• Triceps skinfold thickness<br>• Subscapular skinfold thickness<br>• Head circumference<br>• Mid Upper Arm Circumference (MUAC)
24 to 36 weeks	OGTT (Fasting plasma glucose, 2-h postprandial plasma glucose)<br>Haemoglobin
At delivery	Delivery details<br>Mother’s weight<br>Cause of death, if any<br>Random Blood Sugar for GDM women<br>Initiation of breastfeeding and feeding practice<br>Assessment of depressive symptoms (using EPDS)
Post-delivery -14 weeks, 1,2,3,4 years	Health status<br>Medications/supplements taken<br>Body Mass Index (BMI)<br>Blood pressure<br>Public health facility investigation reports<br>Assessment of depressive symptoms (using EPDS)<br>Social support<br>Anthropometry measurements<br>• Weight<br>• Biceps skinfold<br>• Triceps skinfold<br>• Subscapular skinfold
Child*: At birth, 14th week, 1,2,3, 4 years*	Body Mass Index (BMI)<br>Anthropometry measurements<br>• Weight<br>• Length<br>• Crown-rump length (birth to 1 year)<br>• Head circumference<br>• Chest circumference

For peer review only - http://bmjopen.bmj.com/site/about/guidelines.xhtml

	• Waist circumference<br>• Hip circumference<br>• Mid upper arm circumference<br>• Biceps skinfold thickness<br>• Triceps skinfold thickness<br>• Subscapular skinfold thickness<br>Infant data & neonatal problems<br>Morbidity/disease/Neonatal Intensive Care Unit/Paediatric Intensive Care Unit admissions<br>Immunization records<br>Medications & supplement use<br>Diet<br>Milk or formula feeding<br>Introduction of complementary food and reason<br>Cessation of breastfeeding and reason
14 weeks, 1,2,3, 4 years	Trivandrum Developmental Screening Chart
2 years	Toddler feeding practice<br>Modified Checklist for Autism in Toddlers<br>Physical activity

peer review only

We use the support from frontline health workers in the community to remind and help track the unavailable participants for follow-up visits. Additionally, the research staff regularly call the participants for reminders regarding the follow-up schedule, and if required, also do house visits (after prior permission). These additional efforts are done for participants who cannot visit the public health facility during the defined period. To make it easier for participants, we also schedule follow-up visits in the primary health centres and *Anganwadi* centres near their residence. We have also examined the role of an interactive voice-based response system (IVRS) and an information workshop in improving the follow-up rates in these women (29).

Through IVRS, women received a 2–3 minutes call to inform them about OGTT test dates, collect their laboratory test reports, and remind them of their follow-up visits. The IVRS also provided health information on Gestational Diabetes Mellitus (GDM), breastfeeding, and immunization.

For peer review only - http://bmjopen.bmj.com/site/about/guidelines.xhtml

A Mother and Baby Affairs (MBA) workshop or antenatal workshop and counselling for parents were conducted. This included a lunch for the participant and her family (husband, and children, if any). Generally, this is called "*Seemantha*" in *Kannada*, similar to a baby shower in other parts of the world. The workshop included a brief talk by health professionals on the importance of GDM screening and management and other antenatal and postnatal topics. A health-related quiz competition was followed by gifts distribution to winners. Role-plays were done on personal hygiene, the importance of having nutritional food, delivering at the hospital, precautions at the time of delivery, caring for the baby, feeding of colostrum, homemade baby food, immunization, and family planning measures.

Storage of blood samples for future analysis

At 24 to 36 weeks, during the collection of blood for OGTT, we collect 11 ml venous blood samples in the fasting state and 2 ml for postprandial blood glucose analysis. The blood is collected in three vacutainers; plain (6 ml for storage), Ethylenediaminetetraacetic acid (EDTA) (3 ml for Hemoglobin analysis) and sodium fluoride vacutainers (2 ml each for glucose analysis in fasting and postprandial), respectively. Blood samples are centrifuged and transferred to cryovials within an hour of collecting in cool boxes to a single central laboratory for assays with external quality assurance mechanisms.

Measurements

We perform anthropometric measurements of mothers at the baseline and during subsequent follow-up visits. The weight is measured using Tanita weighing scale, height using the SECA 213 portable stadiometer. The mother's blood pressure (BP) is measured using the automated digital device (Omron Digital BP measuring device). The anthropometric measurements of the baby are recorded within 72 hours of delivery. Newborn anthropometry was performed using SECA 354 Weighing Scale and SECA 417

For peer review only - http://bmjopen.bmj.com/site/about/guidelines.xhtml

Infantometer. We measure the head, mid-upper arm (MUAC), chest, and waist circumferences in both the mother and child using Chasmors body circumference tape. The sum of skinfold thickness (biceps, triceps, and subscapular) (SFT) is measured on the left side of the body using the Holtain Calipers (Holtain Ltd, UK).

Quality control

Research assistants were trained and certified by St. Johns Research Institute, Bengaluru, in anthropometric measurements. Strict protocols are followed to maintain accuracy, with the addition of inter-and intra-observer reliability of measurements assessed at the outset followed by annual certification by the same institute. Trained phlebotomists collect venous blood for laboratory investigations. Biochemical assays are conducted at a central nationally-accredited laboratory with internal and external quality checks. Calibration of all the equipments is done every month using prescribed guidelines, with a calibration log maintained by the research staff and supervised by the principal investigator of the study.

Data management and analysis

Data is entered on android tablets regularly using the application specifically designed for the study. The details of the development and use are published elsewhere (30). The primary data collected is exported through MS Excel 2010 and checked periodically by senior team members for data entry errors and missing information. For this paper, the exported data was then cleaned, and explorative data analysis was done to understand and summarize the variables involved. The descriptive analysis was performed using SPSS version 23. The modified EPDS scores cut-off for defining symptoms suggestive of depression was 13 based on a previous study in Karnataka(31), and social support scores were categorized based on the cut-off score of 24 and above as good(≥24) and poor(<24)(32). Adiposity (mother and child) and

For peer review only

For peer review only - http://bmjopen.bmj.com/site/about/guidelines.xhtml

gestational age (child) weight were classified based on percentiles - using 90th percentile as the cut-off value. Adiposity was considered based on the total sum of skinfold thickness (using biceps, triceps and subscapular thickness). At the same time, weight for gestational age was classified considering the weight of children at each gestational age, adjusted for parity and gender(33). The birth weight of children was categorized based on the WHO standards, and pre-term births were defined as gestational age less than 37 weeks (34). Anaemia in pregnant women was categorized on the WHO criteria based on haemoglobin concentration: Anemic when <110 g/L at sea level, and no anemia when above 100g/L. (35) Hypertension among participants was categorized into: Normal (<120/80 mmHg), Pre-hypertension (120-139/80-89 mmHg), Stage 1 hypertension (140-159/90-99 mmHg), and Stage 2 hypertension (≥160/100 mmHg)(36). Body mass index (BMI) of the participants was categorized based on the WHO Asian criteria: Underweight (<18.5), Normal (18.5-22.9), Overweight (23.0-24.9), and Obese(>25.0)(37). Socioeconomic status was categorized using the modified *Kuppuswamy* socioeconomic status scale 2017 that classifies socioeconomic status as: lower, middle, and upper classes(38).

For peer review only

For peer review only - http://bmjopen.bmj.com/site/about/guidelines.xhtml

Findings to date and discussion

Among 5694 pregnant women, 85.3% (n=4862) were included in the study based on the eligibility criteria and willingness to participate. A total of 67% of women underwent OGTT (n=3260) as per the schedule between 24 to 36 weeks, with a completion rate of 90.9% (n=2962) (Fig. 1). At the baseline, the mean age of the pregnant women was 24.25 ±4.06 years, with 66% between the age group of 18 to 25 years. Most pregnant women had high school education (66%; n=437). More than 90% of women were homemakers (90.0%; n=4446), while most of their spouses were unskilled workers (46.7%; 2237%). Using the modified *Kuppuswamy* scale, most participants were categorized as lower socioeconomic groups (54.6%; n= 2614). (Table 2)

We found the prevalence of Gestational diabetes mellitus in these women was 14.3% with 95% Confidence Interval (CI): 0.13-0.16 (n=424/2962). Among the recruited participants, 265 were pre-hypertensive (7.1%; 95% CI:0.69 - 0.72), and 15 were hypertensive (0.4%; 95% CI: 0.0020 - 0.0060), while 44.5% of the women were anemic (n=1377; 95% CI: 0.42 - 0.46). Using the cut off value of 13 for EPDS, we found that 6.2% of women (n=289) had depressive symptoms indicative of depression during pregnancy, and symptoms of postnatal depression were present in 16.8% of women (n=471). The mean gestational age at delivery was 38.7 ± 3.02 weeks. More than half of the newborns were males (51.9%; n=1717), 43.8% of the infants were delivered by caesarean section (n=1456), and 91.2% infants were full-term (n=4432). (Table 2)

For peer review only - http://bmjopen.bmj.com/site/about/guidelines.xhtml

Table 2: Baseline characteristics of eligible pregnant mothers and children in the MAASTHI birth cohort, Bengaluru, 2016-2021

**Maternal characteristics**	
**Socio-demographic characteristics**	**n (%)**
**Age (years) (N=4862)**	
Mean age± SD	24.25 ± 4.06
18-25	3211 (66.0)
26-35	1598 (32.9)
36-45	53 (1.1)
**Religion (N=4862)**	
Hindu	2454 (50.5)
Muslim	2142 (44.1)
Others	266 (5.5)
**Consanguineous marriage (N=4788)**	1157 (24.2)
**Participant's education (N=4788)**	
Illiterate	169 (3.5)
Primary school	284 (5.9)
Middle school	609 (12.7)
High school	2091 (43.7)
PUC or diploma	1072 (22.4)
Graduate and above	563 (11.8)
**Husband's education (N=4788)**	
Illiterate	426 (8.9)
Primary school	461 (9.6)
Middle school	724 (15.1)
High school	1956 (40.9)
PUC or diploma	793 (16.6)
Graduate and above	422 (8.8)
Do not know	6 (0.1)
**Participant's occupation (N=4788)**	
Homemaker	4446 (92.9)
Unskilled worker	168 (3.5)
Semiskilled worker	76 (1.6)
Skilled worker	85 (1.8)
Professional	13 (0.3)
**Husband's occupation (N=4788)**	
Unemployed	17 (0.4)
Unskilled worker	2237 (46.7)
Semiskilled worker	1535 (32.1)
Skilled worker	860 (18.0)
Professional	139 (2.9)
**Socio-economic status (N=4788)**	
Lower class	2614 (54.6)
Middle class	2145 (44.8)
Upper class	29 (0.6)
**Chronic conditions with 95% CI of prevalence estimates**	
**Hypertension (N=3719)**	

For peer review only

For peer review only - http://bmjopen.bmj.com/site/about/guidelines.xhtml

Normal<br>Pre hypertension<br>Stage 1 hypertension	3429 (92.2) (95% CI: 0.91 - 0.93)<br>265 (7.1) (95% CI: 0.69 - 0.72)<br>15 (0.4) (95% CI: 0.0020 - 0.0060)
**GDM during current pregnancy (N=2962)**	424 (14.3) (95% CI: 0.13 - 0.16)
**Anemia (N=3097)**	1377 (44.5) (95% CI: 0.42 - 0.46)
**Obstetrical characteristics (N=4778)**	
**Gravida**	
Primigravida<br>Multigravida	1920 (40.2)<br>2858 (59.8)
**Parity**	
Nulliparous<br>Multiparous	2178 (45.6)<br>2600 (54.4)
**Mean Gestational age at delivery (week) (N=3324)**	38.700 ± 3.02
**Depressive symptoms and social support**	
EPDS score at pregnancy (>13 cut-off) (N=4635)<br>Social support score above 24 (N=4643)<br>EPDS score at delivery (>13 cut-off) **(N=2801)**	289 (6.2)<br>1520 (32.7)<br>471 (16.8)
**Health status of mothers at delivery (N=3324)**	
Healthy<br>Diseased<br>Death	3054 (92.0)<br>259 (7.8)<br>7 (0.2)
**Child characteristics n (%)**	
**Health status at birth (N=3310)**	
Healthy<br>Diseased<br>Death	3188 (96.3)<br>49 (1.5)<br>73 (2.2)
**Preterm deliveries (N=4862)**	430 (8.8)
**Birth weight (N=4862)**	
Underweight<2500g<br>Normal 2500 g to 3500 g<br>Overweight >3500g	384 (7.9)<br>1625 (33.4)<br>2853 (58.7)
**Birth weight according to Gestational Age (N=4862)**	
Small for Gestational Age (SGA)<br>Mean ± SD weight for SGA (kg)<br>Large for Gestational Age (LGA)<br>Mean ± SD weight for LGA (kg)	159 (3.3)<br>2.13 ± 0.26<br>523 (10.8)<br>4.10 ± 0.72
**Delivery type (N=3324)**	
Vaginal delivery<br>Caesarean section	1868 (56.2)<br>1456 (43.8)
**Sex (N=3309)**	
Male<br>Female	1717 (51.9)<br>1592 (48.1)
**Baby cried soon after delivery (N=3310)**	3030 (91.5)
**Colostrum fed to the baby (N=2915)**	2562 (87.9)
**Breast milk fed as first food to the baby (N=2915)**	1932 (66.3)
**Initiation of breast feeding after delivery (N=2890)**	
Within an hour<br>Between 1-24 hour<br>After 24 hour	1227 (42.4)<br>1538 (53.2)<br>125 (4.4)

For peer review only - http://bmjopen.bmj.com/site/about/guidelines.xhtml

Table 3 and 4 summarizes the anthropometric measurements of the participants (mothers) and their offspring during each phase of the study period respectively. Categorization is done based on tertiles - 10th, 10 – 90th, and 90th percentiles. Each table includes mean and standard deviation (SD) values for the measurements as described in the analysis, along with the frequency and proportion of mother and child in each tertile. We found that among the mothers above the 90th percentile for weight, 9.9% were obese at delivery and subsequent follow-ups. They weighed an average of 82.74 kg as compared to 83.84 kg at the 3-years follow-up. During the period from delivery to the 3-year follow up, the average SFT in women with obesity (>90th percentile of SFT) changed from 76.6 mm (9.7%) to 75.46 mm (9.9%) in comparison to women with a normal range of SFT (10 to 90th percentile) in whom it changed from 46.54 mm (80.6%) to 49.79 mm (80.2%) (Table 3).

For peer review only

For peer review only - http://bmjopen.bmj.com/site/about/guidelines.xhtml

Table 3: Anthropometry of the mother at different follow-up periods of the MAASTHI birth cohort, Bengaluru, 2016-21

Anthropometric measurement		During Pregnancy (N=3734)		14th week follow up (N=2146)		1 year follow up (N=1255)		2 year follow up (N=801)		3 year follow up (N=393)	
		n (%)	Mean (SD)	n (%)	Mean (SD)	n (%)	Mean (SD)	n (%)	Mean (SD)	n (%)	Mean (SD)
Weight	<10th percentile	367 (9.8)	41.88 (2.54)	214 (10.0)	40.35 (2.73)	124 (9.9)	38.96 (2.21)	80 (10.0)	40.25 (2.91)	39 (9.9)	40.41 (2.04)
	10-90th percentile	2996 (80.2)	58.09 (7.60)	1720 (80.1)	56.90 (7.76)	1006 (80.2)	56.54 (8.00)	642 (80.1)	59.25 (8.18)	315 (80.2)	59.27 (8.31)
	>90th percentile	371 (9.9)	82.74 (7.07)	212 (9.9)	81.27 (6.56)	124 (9.9)	81.79 (7.02)	79 (9.9)	85.86 (8.51)	39 (9.9)	83.84 (7.07)
Waist circumference	<10th percentile	-	-	194 (9.6)	61.51 (3.71)	124 (9.9)	60.18 (2.97)	80 (10.0)	61.04 (2.71)	38 (9.7)	60.27 (3.65)
	10-90th percentile	-	-	1635 (80.8)	77.96 (7.51)	1006 (80.2)	77.43 (7.61)	641 (80.0)	79.87 (8.41)	319 (81.2)	79.01 (8.06)
	>90th percentile	-	-	195 (9.6)	99.46 (6.23)	125 (10)	98.99 (6.15)	80 (10.0)	103.59 (6.00)	36 (9.2)	98.82 (4.07)
MUAC	<10th percentile	358 (9.6)	20.01 (1.15)	190 (8.8)	20.57 (1.02)	124 (9.9)	20.39 (1.19)	74 (9.2)	20.59 (1.44)	39 (9.9)	20.44 (1.41)
	10-90th percentile	3002 (80.4)	25.78 (2.55)	1744 (81.2)	26.42 (2.52)	1012 (80.6)	26.74 (2.62)	649 (81.0)	27.65 (2.68)	315 (80.2)	27.30 (2.65)
	>90th percentile	372 (10.0)	33.54 (2.11)	213 (9.9)	34.03 (2.08)	119 (9.5)	43.43 (2.05)	78 (9.7)	35.30 (2.05)	39 (9.9)	34.44 (1.66)
Total SFT	<10th percentile	361 (9.7)	25.21 (2.91)	213 (9.9)	24.72 (3.51)	124 (9.9)	22.44 (3.38)	79 (9.9)	25.37 (4.19)	39 (9.9)	24.19 (4.12)

For peer review only

For peer review only - http://bmjopen.bmj.com/site/about/guidelines.xhtml

	10-90th percentile	3008 (80.6)	46.54 (10.05)	1723 (80.3)	46.84 (9.59)	1007 (80.2)	45.93 (10.17)	641 (80.1)	50.69 (10.20)	315 (80.2)	49.79 (10.17)
	>90th percentile	363 (9.7)	76.60 (8.31)	210 (9.8)	73.83 (6.75)	125 (10.0)	73.92 (6.04)	80 (10.0)	79.55 (8.15)	39 (9.9)	75.46 (7.00)
Hip Circumference	<10th percentile	-	-	199 (9.8)	79.39 (4.85)	121 (9.6)	77.52 (4.00)	80 (10.0)	78.71 (5.93)	39 (9.9)	80.33 (3.29)
	10-90th percentile	-	-	1624 (80.2)	94.99 (6.71)	1013 (80.7)	94.75 (7.06)	642 (80.1)	97.18 (7.16)	315 (80.2)	96.69 (6.89)
	>90th percentile	-	-	201 (9.9)	115.35 (5.47)	121 (9.6)	115.47 (5.71)	79 (9.9)	118.56 (5.75)	39 (9.9)	115.63 (3.29)
Height	<10th percentile	368 (9.9)	144.15 (2.27)	-	-	-	-	-	-	-	-
	10-90th percentile	2993 (80.2)	153.81 (3.80)	-	-	-	-	-	-	-	-
	>90th percentile	373 (10.0)	164.16 (2.77)	-	-	-	-	-	-	-	-
Head circumference	<10th percentile	329 (8.8)	49.46 (1.09)	-	-	-	-	-	-	-	-
	10-90th percentile	3080 (82.5)	52.69 (1.15)	-	-	-	-	-	-	-	-
	>90th percentile	324 (8.7)	56.08 (1.00)	-	-	-	-	-	-	-	-

Anthropometric measurements of children showed that the sum of skinfold thickness ranged from 22.37 mm to 32.6 mm from birth to the 3-year follow-up for children above the 90th percentile compared to 13.76 mm to 22.87 mm, respectively in children between 10 to the 90th percentile. Birth of a high

For peer review only

For peer review only - http://bmjopen.bmj.com/site/about/guidelines.xhtml

proportion of obese children (weight above 90th percentile), i.e., 9.7%, was noted in our study (Table 4). We also noted that 7.9% of children born were low birth weight (2500 gms or less), and 8.8% (n=430) were born pre-term. A high proportion of 10.8% (n=523) of children born were large for gestational age (LGA) in our cohort, and 3.3% were small for gestational age (SGA) (n=159). The mean weight of children who were SGA at birth was 2.13 ± 0.26 kg and was 4.10 ± 0.72 kg for children with LGA.

For peer review only

For peer review only - http://bmjopen.bmj.com/site/about/guidelines.xhtml

291 **Table 4: Anthropometry of the child at different follow-up periods of the MAASTHI birth cohort,**
292 **Bengaluru, 2016-21**

**Anthropometric measurement**		**At birth**		**14 week follow up**		**1 year follow up**		**2 year follow up**		**3 year follow up**	
		**n (%)**	**Mean (SD)**	**n (%)**	**Mean (SD)**	**n (%)**	**Mean (SD)**	**n (%)**	**Mean (SD)**	**n (%)**	**Mean (SD)**
Weight	<10th percentile	243 (9.8)	2.14 (0.21)	182 (8.5)	4.31 (0.39)	122 (9.7)	6.84 (0.38)	74 (9.0)	8.56 (0.62)	41 (10.0)	9.80 (0.64)
	10-90th percentile	2003 (80.5)	2.98 (0.41)	1761 (82.4)	6.04 (0.72)	1010 (80.5)	8.74 (0.82)	670 (81.5)	10.73 (0.90)	333 (81.2)	12.30 (0.95)
	>90th percentile	242 (9.7)	4.78 (0.74)	195 (9.1)	8.37 (0.73)	122 (9.7)	11.43 (0.85)	78 (9.5)	13.41 (0.77)	36 (8.8)	14.74 (0.28)
CRL	<10th percentile	233 (9.4)	27.37 (2.01)	213 (10.0)	33.21 (1.89)	114 (9.9)	37.28 (2.48)	-	-	-	-
	10-90th percentile	2020 (81.5)	32.41 (2.01)	1719 (80.6)	40.52 (2.40)	926 (80.2)	45.31 (2.36)	-	-	-	-
	>90th percentile	226 (9.1)	39.29 (2.21)	202 (9.5)	47.66 (2.79)	114 (9.9)	53.44 (4.00)	-	-	-	-
Length	<10th percentile	248 (10.0)	43.69 (1.72)	206 (9.6)	55.26 (2.22)	125 (10.0)	65.76 (6.28)	79 (9.6)	74.64 (3.09)	41 (10.0)	83.16 (3.42)
	10-90th percentile	2009 (80.8)	49.38 (2.28)	1720 (80.5)	62.66 (2.74)	1003 (80.2)	73.79 (2.65)	664 (80.8)	83.17 (2.92)	329 (80.2)	90.78 (2.77)
	>90th percentile	230 (9.2)	58.06 (2.81)	211 (9.9)	70.99 (2.24)	123 (9.8)	81.68 (2.78)	79 (9.6)	91.61 (2.23)	40 (9.8)	97.89 (1.65)
Head Circumference	<10th percentile	242 (9.7)	30.30 (1.07)	199 (9.3)	36.30 (2.05)	102 (8.1)	40.39 (1.79)	81 (9.9)	42.49 (1.45)	39 (9.5)	43.96 (0.87)
	10-90th percentile	2001 (80.4)	33.66 (1.33)	1740 (81.4)	40.07 (1.33)	1033 (82.4)	44.23 (1.16)	666 (81.3)	46.16 (1.25)	332 (81.0)	47.10 (1.09)
	>90th percentile	245 (9.8)	38.10 (1.25)	199 (9.3)	43.97 (0.96)	119 (9.5)	47.41 (1.33)	72 (8.8)	50.81 (4.86)	39 (9.5)	50.27 (1.48)
Chest circumference	<10th percentile	246 (9.9)	28.10 (1.19)	198 (9.3)	35.67 (1.02)	125 (10.0)	39.73 (1.89)	69 (8.4)	42.54 (1.41)	38 (9.3)	44.57 (0.87)
	10-90 percentile	2014 (80.9)	32.24 (1.73)	1740 (81.4)	40.12 (1.71)	1017 (81.1)	44.35 (1.58)	673 (81.8)	46.78 (1.58)	333 (81.2)	48.56 (1.69)
	>90th percentile	228 (9.2)	38.38 (1.83)	200 (9.4)	45.08 (1.27)	112 (8.9)	49.15 (1.48)	81 (9.8)	56.49 (1.64)	39 (9.5)	53.0 (1.04)
Waist circumference	<10th percentile	235 (9.4)	24.81 (1.17)	182 (8.5)	33.14 (1.46)	2 (0.2)	29.30 (1.69)	77 (9.3)	40.17 (1.64)	41 (10.0)	41.93 (1.20)
	10-90 percentile	2008 (80.7)	30.66 (2.47)	1747 (81.7)	39.28 (2.29)	1132 (90.3)	41.95 (2.86)	667 (80.9)	45.57 (1.98)	329 (80.2)	46.94 (2.09)
	>90th percentile	245 (9.8)	38.24 (1.87)	209 (9.8)	45.37 (1.58)	119 (9.5)	49.02 (1.83)	80 (9.7)	51.39 (1.96)	40 (9.8)	53.27 (1.40)
Hip Circumference	<10th percentile	211 (8.5)	23.67 (0.93)	211 (9.9)	32.05 (1.49)	4 (0.3)	30.75 (2.12)	79 (9.6)	39.71 (1.47)	39 (9.5)	41.70 (4.69)
	10-90 percentile	2037 (81.9)	28.91 (2.44)	1717 (80.3)	39.05 (2.85)	1125 (89.7)	42.17 (3.06)	669 (81.2)	46.23 (2.51)	332 (81.0)	48.71 (2.50)

For peer review only - http://bmjopen.bmj.com/site/about/guidelines.xhtml

	>90th percentile	240 (9.6)	37.32 (2.68)	210 (9.8)	46.42 (1.60)	125 (10.0)	50.0 (1.97)	76 (9.2)	53.47 (1.87)	39 (9.5)	55.21 (1.31)
MUAC	<10th percentile	191 (7.7)	7.86 (0.58)	206 (9.6)	10.58 (0.76)	124 (9.9)	11.25 (0.96)	78 (9.5)	11.65 (0.82)	39 (9.5)	12.28 (1.47)
	10-90 percentile	2074 (83.4)	9.81 (0.76)	1748 (81.8)	12.88 (0.82)	1015 (80.9)	13.77 (0.84)	665 (80.8)	14.15 (0.80)	333 (81.2)	14.57 (0.73)
	>90th percentile	223 (9.0)	12.48 (0.77)	183 (8.6)	15.31 (0.70)	115 (9.2)	16.25 (1.52)	80 (9.7)	16.72 (1.03)	38 (9.3)	17.76 (5.78)
Total SFT	<10th percentile	223 (9.0)	9.27 (0.78)	196 (9.2)	14.25 (1.20)	119 (9.5)	14.36 (0.98)	78 (9.5)	14.94 (1.20)	40 (9.8)	15.53 (1.06)
	10-90 percentile	2005 (81.0)	13.76 (2.14)	1723 (81.2)	20.80 (2.61)	1006 (80.5)	19.98 (2.47)	670 (81.4)	21.58 (3.05)	332 (81.0)	22.87 (3.41)
	>90th percentile	246 (9.9)	22.37 (3.25)	204 (9.6)	29.45 (3.31)	124 (9.9)	28.02 (2.74)	75 (9.1)	32.46 (3.74)	38 (9.3)	32.60 (3.28)

Strengths and limitations

The high prevalence of GDM, as evidenced by the ongoing birth cohort study, may provide evidence for a policy decision to scale up screening and management of GDM in all the public health facilities. As there is no unanimity regarding appropriate guidelines for diagnosing GDM for the Indian population, our findings can provide specific evidence regarding exact cut-off points to be used for the diagnosis, above which it may have an association with adverse maternal and childhood outcomes. Our secondary objective, based on the psychosocial environment of the mother and the huge burden of depression among mothers, draws attention to the importance of mental health screening as part of routine antenatal care. As our study is focused on maternal antecedents of childhood obesity such as nutritional, psychosocial and environmental factors and the outcome being childhood obesity, our findings provide clear evidence for designing and developing effective interventions for the prevention of childhood obesity.

We found a high number of children born LGA. Additionally, the prevalence of obesity at birth and subsequent follow-ups was also high (above 9%). If unchanged, the modifiable factors and GDM will continue the vicious cycle of afflicting subsequent generations (39). The results from our cohort will continue to inform and shape policies regarding screening, appropriate care and management for persons

For peer review only

affected with GDM and perinatal depression (40-42). Also, from studying the ecosystem of NCDs in growing economies, our longitudinal study of vulnerable women and children provides many research opportunities. It has great potential to address scientific and public health questions.

MAASTHI is a well-established cohort study documenting the life course trajectory in the genesis of NCDs. We are documenting important exposure contrasts experienced by women very early on in their pregnancy and continue to document the outcomes across the process of pregnancy, childbirth and early childhood, including assessing important milestones of the child. In India, public health facilities are the preferred choice for nearly 50% of the entire Indian population (43); and nearly every woman from the lower social strata seeks care from public health facilities. Through the concerted efforts of governments, donors, and health service providers, maternal and infant mortality rates have reduced drastically in the past decade in the country. Despite these efforts, maternal and neonatal mortality and morbidity are relatively high compared to other LMICs such as Bangladesh and Sri Lanka (44-46). Our findings revealed an unusually high burden of GDM and maternal obesity similar to earlier studies (47-50) for women between the age group of 18 to 45 years. Our cohort study will inform policies in achieving further reductions of maternal and fetal mortality and morbidity. For example, achieving the desired level of glycemic control during pregnancy can reduce several maternal and fetal adverse effects (51). Early screening and timely management of GDM can prevent disastrous outcomes for mother and child and further generations.

The strength of MAASTHI is that data pertains to the pregnant women and children belonging to lower- and middle-income socioeconomic status-seeking healthcare from the public health facilities. The strength of the cohort is the strong representation of minority groups and vulnerable sections of the society that utilizes public health facilities. Also, this is probably the only pregnancy cohort recruited from public health facilities in India. Although the acronym suggests the transgenerational role of glucose and

For peer review only - http://bmjopen.bmj.com/site/about/guidelines.xhtml

insulin, the study was conceptualized to include important maternal determinants of underweight and childhood obesity. The comprehensive exposure assessments in MAASTHI include the role of nutritional, psychosocial, and, recently more relevant - air pollution throughout pregnancy and different time points of the life course. Attention to obtaining high-quality data by maintaining stringent quality checks for reproducible measurements and minimal errors is another major strength of this study. Moreover, blood aliquots are stored in the biorepository for future studies on childhood obesity.

Operational issues in terms of incomplete OGTT and loss-to-follow-up after delivery are the limitations of conducting a study in public health facilities. In the absence of a registry or defined population for health care services, this limitation will continue to affect implementing cohort studies in public health facilities. Periods of prolonged fasting (previous night) coupled with the issue of gastritis in pregnant women are other factors responsible for incomplete OGTT. As with most research during COVID-19, our cohort was also subject to the issues of data collection. Our research staff had to resolve the inability of physical contact for face-to-face interviews through telephonic interviews. However, no physical meetings meant the complete stop to anthropometric measurements for mother-child dyads.

By setting up and following up this cohort, we can inform the design and implement interventions focusing on health promotion and disease prevention, address undernutrition and overweight epidemics, and thereby contribute to reducing the NCD disease profile in the country.

For peer review only

For peer review only - http://bmjopen.bmj.com/site/about/guidelines.xhtml

Collaboration

The MAASTHI prospective cohort will continue to collect data on various exposures and maternal and child health related outcomes. For more information, refer to the web site: maasthi.com. MAASTHI encourages proposals from outside investigators: Proposals involving the use of existing data or the collection of new data. Researchers interested in collaboration are invited to submit requests for MAASTHI clinical data through a research proposal to birthcohort@iiphh.org

For peer review only

For peer review only - http://bmjopen.bmj.com/site/about/guidelines.xhtml

Abbreviations

ANC: Ante-natal check-up; BBMP: Bruhat Bengaluru Mahanagara Palike; BMI: Body Mass Index; EDTA: Ethylene Diamine Tetra Acetic acid; CC: Chest circumference; CRL: Crown rump length; CRP: C Reactive Protein, EPDS: Edinburgh Postnatal Depression Scale; GDM: Gestational diabetes mellitus; HAPO: Hyperglycemia and adverse pregnancy outcome study; HC: Head circumference; HIV: Human immunodeficiency virus; IEC: Institutional Ethics Committee; IIPH: Indian Institute of Public Health; IL: interleukin; LMIC: Low- and middle-income countries; LGA: Large for gestational age; LBW: Low birth weight; MAASTHI: Maternal antecedents of adiposity studying the transgenerational role of hyperglycaemia and insulin; MBA: Mother and baby affairs; MCHAT: Modified Checklist for Autism in Toddlers; MUAC: Middle-upper arm circumference; NCD: Non-Communicable Disease; OGTT: Oral glucose tolerance test; SD: Standard Deviation; SFT: Sum of skinfold thickness; SGA: Small for gestational age; T2DM: Type 2 diabetes mellitus; TNF: tumor necrosis factor ; WC: Waist circumference; WHO: World Health Organization

For peer review only

For peer review only - http://bmjopen.bmj.com/site/about/guidelines.xhtml

Acknowledgements

We sincerely thank the Department of Health and Family Welfare, the Government of Karnataka (DoHFW, GoK) and the Bruhat Bengaluru Mahanagara Palike (BBMP) for permitting us to conduct the study and providing constant support. We thank hospitals under DoHFW, GoK, Superintendents, Medical Officers, Doctors, and all the department support staff for supporting an ongoing study. We thank Dr Sumathi Swaminathan and her team at St John's Research Institute for training the Research Assistants in anthropometry measurement. Our sincere thanks to the Welcome Trust, DBT India Alliance, for the support with funding and guidance. Our sincere thanks to Dr. Suresh Shapeti and Mr. T.S. Ramesh for facilitating the administrative support and coordination. We thank all the research team members of MAASTHI for their support in carrying out research activities in the field. We thank all participants for their effort to enroll and continuous participation in the ongoing cohort.

Funding

This work was supported by the Wellcome Trust/DBT India Alliance Fellowship [Grant No. IA/CPHI/14/1/501499] awarded to Giridhara R. Babu. The funding agency had no role in the design and conduct of the study; collection, management, analysis, and interpretation of the data; preparation, review, or approval of the manuscript; or decision to submit the manuscript for publication.

Conflict of interest

The authors declare that they have no conflict of interest.

For peer review only - http://bmjopen.bmj.com/site/about/guidelines.xhtml

Ethics consideration

The study was reviewed and approved by the Institutional ethics committee (IEC) of the Indian Institute of Public Health – Bengaluru (Approval number IIPHHB/TRCIEC/091/2015 dated 13th November 2015). The progress of the study is reviewed by the IEC, and permissions are extended annually thereafter. Informed consent prior to participation in the study was obtained from all voluntarily willing participants. The written informed consent is obtained for participation, follow-up, and permission to publish anonymous data in any report, journal and for assessing the stored samples for study-related hypotheses. The study was conducted in accordance with the Declaration of Helsinki.

Authors contributions

GRB was involved in the conception, design, and funding acquisition. YA, DR, PS, MK, SN performed the experiments. EL, GRB were involved in drafting the paper and revising the draft. SK, GVSM were involved in the critical analysis of the paper. All the authors have approved the final version of the article submitted.

Data Sharing Statement

To discuss our data sharing policy, please contact Giridhara R. Babu at giridhar@phfi.org

For peer review only

For peer review only - http://bmjopen.bmj.com/site/about/guidelines.xhtml

References

1. India : Health of the Nation's States - The India State-level Disease Burden Initiative. 2017.
2. Hales CN, Barker DJ. The thrifty phenotype hypothesis. British medical bulletin. 2001;60:5-20.
3. Mohan V, Sandeep S, Deepa R, Shah B, Varghese C. Epidemiology of type 2 diabetes: Indian scenario. The Indian journal of medical research. 2007;125:217-30.
4. Shaw JE, Sicree RA, Zimmet PZ. Global estimates of the prevalence of diabetes for 2010 and 2030. Diabetes research and clinical practice. 2010;87:4-14.
5. Anjana RM, Pradeepa R, Deepa M, Datta M, Sudha V, Unnikrishnan R, et al. Prevalence of diabetes and prediabetes (impaired fasting glucose and/or impaired glucose tolerance) in urban and rural India: Phase I results of the Indian Council of Medical Research–INdia DIABetes (ICMR–INDIAB) study. Diabetologia. 2011;54:3022-7.
6. Mithal A, Bansal B, Kalra S. Editorial Gestational diabetes in India : Science and society. 2015:155-8.
7. Prasad A. Type 2 diabetes mellitus in young: need for early screening. Indian pediatrics. 2011;48(9):683.
8. Buchanan T, Freinkel N, Lewis NJ, Metzger BE, Akazawa S. Fuel-mediated teratogenesis. Use of D-mannose to modify organogenesis in the rat embryo in vivo. Journal of Clinical Investigation. 1985;75:1927-34.
9. Group THSCR. Hyperglycemia and Adverse Pregnancy Outcomes. New England Journal of Medicine. 2008;358:1991-2002.
10. HAPO Study Cooperative Research Group THSCR. Hyperglycemia and Adverse Pregnancy Outcome (HAPO) Study: associations with neonatal anthropometrics. Diabetes. 2009;58:453-9.
11. Yajnik CS. Nutrient-mediated teratogenesis and fuel-mediated teratogenesis: two pathways of intrauterine programming of diabetes. International journal of gynaecology and obstetrics: the official organ of the International Federation of Gynaecology and Obstetrics. 2009;104 Suppl 1:S27-31.
12. Neel JV. Diabetes mellitus: a "thrifty" genotype rendered detrimental by "progress"? American journal of human genetics. 1962;14:353-62.
13. Freinkel N. Banting Lecture 1980. Of pregnancy and progeny. Diabetes. 1980;29:1023-35.
14. Hattersley AT, Tooke JE. The fetal insulin hypothesis: an alternative explanation of the association of low bir thweight with diabetes and vascular disease. The Lancet. 1999;353:1789-92.
15. Fetita L-S, Sobngwi E, Serradas P, Calvo F, Gautier J-F. Consequences of Fetal Exposure to Maternal Diabetes in Offspring. The Journal of Clinical Endocrinology & Metabolism. 2006;91:3718-24.
16. Fall CHD, Stein CE, Kumaran K, Cox V, Osmond C, Barker DJP, et al. Size at birth, maternal weight, and Type 2 diabetes in South India. Diabetic Medicine. 1998;15:220-7.
17. Gillman MW, Rifas-Shiman S, Berkey CS, Field AE, Colditz GA. Maternal gestational diabetes, birth weight, and adolescent obesity. Pediatrics. 2003;111:e221-6.

For peer review only - http://bmjopen.bmj.com/site/about/guidelines.xhtml

18. Sobngwi E, Boudou P, Mauvais-Jarvis F, Leblanc H, Velho G, Vexiau P, et al. Effect of a diabetic environment in utero on predisposition to type 2 diabetes. The Lancet. 2003;361:1861-5.

19. Yadav S, Arokiasamy P. Understanding epidemiological transition in India. Global Health Action. 2014;7(SUPP.1):1--14.

20. Brisbois TD, Farmer AP, McCargar LJ. Early markers of adult obesity: a review. obesity reviews. 2012;13(4):347-67.

21. Targets WWGN. 2025: Low birth weight policy brief. Geneva: WHO. 2014.

22. Vir SC. Improving women's nutrition imperative for rapid reduction of childhood stunting in South Asia: coupling of nutrition specific interventions with nutrition sensitive measures essential. Maternal & child nutrition. 2016;12:72-90.

23. Feldman PJ, Dunkel-Schetter C, Sandman CA, Wadhwa PD. Maternal social support predicts birth weight and fetal growth in human pregnancy. Psychosomatic medicine.62:715-25.

24. Diego MA, Jones NA, Field T, Hernandez-Reif M, Schanberg S, Kuhn C, et al. Maternal Psychological Distress, Prenatal Cortisol, and Fetal Weight. Psychosomatic Medicine. 2006;68:747-53.

25. Babu GR, Garadi L, Murthy GVS, Kinra S. Effect of hyperglycaemia in pregnancy on adiposity in their infants in India : a protocol of a multicentre cohort study. 2014:1-8.

26. Babu GR, Murthy GVS, Deepa R, Kumar HK, Karthik M. Maternal antecedents of adiposity and studying the transgenerational role of hyperglycemia and insulin (MAASTHI): a prospective cohort study Protocol of birth cohort at Bangalore , India. BMC pregnancy and childbirth. 2016:1-9.

27. Anand SS, Vasudevan A, Gupta M, Morrison K, Kurpad A, Teo KK, et al. Rationale and design of South Asian Birth Cohort (START): a Canada-India collaborative study. BMC Public Health. 2013;13:79.

28. Organization WH. Diagnostic criteria and classification of hyperglycaemia first detected in pregnancy: A World Health Organization Guideline. Diabetes research and clinical practice. 2014;103:341-63.

29. Babu GR, Karthik M, Ravi D, Ana Y, Shriyan P, Hasige KK, et al. What makes the pregnant women revisit public hospitals for research? Participant engagement and retention trial in a public hospital (PERTH): an RCT protocol. BMC pregnancy and childbirth. 2018;18(1):369.

30. Lobo E, Vivekananda P, Deepa R, Babu GR. Development, design, and experience related to Android application-based data collection for the MAASTHI cohort study in India. RGUHS National Journal of Public Health. 2020;3(1):3 - 11.

31. Fernandes MC, Srinivasan K, Stein AL, Menezes G, Sumithra R, Ramchandani PG. Assessing prenatal depression in the rural developing world: a comparison of two screening measures. Archives of women's mental health. 2011;14(3):209-16.

32. Anand SS, Vasudevan A, Gupta M, Morrison K, Kurpad A, Teo KK, et al. Rationale and design of South Asian birth cohort (START): a Canada-India collaborative study. BMC public health. 2013;13(1):1-13.

33. Kumar VS, Jeyaseelan L, Sebastian T, Regi A, Mathew J, Jose R. New birth weight reference standards customised to birth order and sex of babies from South India. BMC pregnancy and childbirth. 2013;13:1.

34. World Health Organization. ICD 10 international statistical classification of diseases and related health problems tenth revision, 2. Geneva: WHO. 2004.

For peer review only

For peer review only - http://bmjopen.bmj.com/site/about/guidelines.xhtml

35. World Health Organization. Haemoglobin concentrations for the diagnosis of anaemia and assessment of severity. Vitamin and Mineral Nutrition Information System. 2011; (WHO/NMH/NHD/MNM/11.1).

36. Program NHBPE. Classification of Blood Pressure-The Seventh Report of the Joint National Committee on Prevention, Detection, Evaluation, and Treatment of High Blood Pressure. US Department of Health And Human Services, National Institutes of Health, National Heart, Lung, and Blood Institute. 2004.

37. Misra A, Chowbey P, Makkar B, Vikram N, Wasir J, Chadha D, et al. Consensus statement for diagnosis of obesity, abdominal obesity and the metabolic syndrome for Asian Indians and recommendations for physical activity, medical and surgical management. Japi. 2009;57(2):163-70.

38. Singh T, Sharma S, Nagesh S. Socio-economic status scales updated for 2017. Int J Res Med Sci. 2017;5(7):3264-7.

39. Catalano PM. Obesity and pregnancy—the propagation of a viscous cycle? : Oxford University Press; 2003.

40. Fisher J, Cabral de Mello M, Patel V, Rahman A, Tran T, Holton S, et al. Prevalence and determinants of common perinatal mental disorders in women in low- and lower-middle-income countries: a systematic review. Bulletin of the World Health Organization. 2012;90:139-49H.

41. Smith MV, Shao L, Howell H, Lin H, Yonkers KA. Perinatal Depression and Birth Outcomes in a Healthy Start Project. Maternal and Child Health Journal. 2011;15:401-9.

42. Rwakarema M, Premji SS, Nyanza EC, Riziki P, Palacios-Derflingher L. Antenatal depression is associated with pregnancy-related anxiety, partner relations, and wealth in women in Northern Tanzania: a cross-sectional study. BMC Women's Health. 2015;15:68.

43. Ministry of Health and Family Welfare GoI. Select Health Parameters : A Comparative Analysis across the National Sample Survey Organization (NSSO) 42 , 52 and 60 Rounds. Ministry of Health and Family Welfare, India. 2007.

44. Sciences IIfP. National Family Health Survey (NFHS-4) 2015-16 India. International Institute for Population Sciences (IIPS) and ICF. 2017:1-192.

45. India RG, editor SRS Bulletin - Sample Registration System. September; 2014.

46. India RGo, editor Special Bulletin on Maternal Mortality in India 2010-12 Sample Registration System2015.

47. Seshiah V, Balaji V, Balaji MS, Sanjeevi CB, Green A. Gestational diabetes mellitus in India. The Journal of the Association of Physicians of India. 2004;52:707-11.

48. Wahi P, Dogra V, Jandial K, Bhagat R, Gupta R, Gupta S, et al. Prevalence of gestational diabetes mellitus (GDM) and its outcomes in Jammu region. The Journal of the Association of Physicians of India. 2011;59:227-30.

49. Anzaku AS, Musa J. Prevalence and associated risk factors for gestational diabetes in Jos, North-central, Nigeria. Archives of Gynecology and Obstetrics. 2013;287:859-63.

50. Ewenighi Chinwe O. 1* NHU, Dimkpa Uche3, Onyeanusi Joel C.1, Nnatuanya Isaac N. 4, Onoh Linus U. M.5, Ezeugwu Uchechukwu 6 , Onoh Gladys O.7, Ibeagi Ogubuike O.8 and Adejumo Babatunde I.9. Prevalence Of Gestational Diabetes Mellitus; Risk Factors Among Pregnant Women. National Journal of Integrated Research in Medicine. 2013;4:56-61.

51. Ogurtsova K, da Rocha Fernandes J, Huang Y, Linnenkamp U, Guariguata L, Cho N, et al. IDF Diabetes Atlas: Global estimates for the prevalence of diabetes for 2015 and 2040. Diabetes research and clinical practice. 2017;128:40-50.

For peer review only - http://bmjopen.bmj.com/site/about/guidelines.xhtml

537
538 **Figure Legend**

539 **Figure 1: Study recruitment and follow-ups in the MAASTHI cohort in India, 2016-2019**

540 The flow chart shows the recruitment to follow-up of participants into the MASSTHI cohort and the
541 reasons for drop-out

For peer review only

For peer review only - http://bmjopen.bmj.com/site/about/guidelines.xhtml

For peer review only

For peer review only - http://bmjopen.bmj.com/site/about/guidelines.xhtml

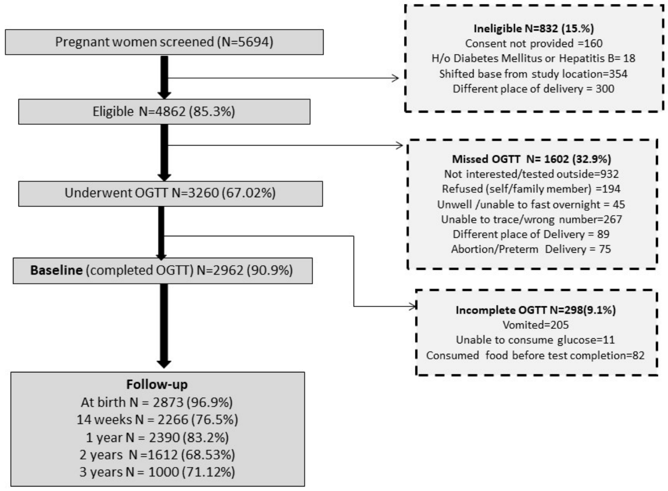

Figure 1

338x190mm (96 x 96 DPI)

Cohort Profile: Maternal antecedents of adiposity and studying the transgenerational role of hyperglycemia and insulin (MAASTHI)

Journal:	*BMJ Open*
Manuscript ID	bmjopen-2022-063794.R1
Article Type:	Cohort profile
Date Submitted by the Author:	19-Aug-2022
Complete List of Authors:	Lobo, Eunice; Public Health Foundation of India<br>Ana, Yamuna; Public Health Foundation of India<br>Deepa, R; Public Health Foundation of India<br>Shriyan, Prafulla; Public Health Foundation of India<br>Sindhu, ND; Public Health Foundation of India<br>Karthik, Maithili; Public Health Foundation of India<br>Kinra, Sanjay; London School of Hygiene & Tropical Medicine<br>Murthy, GVS; Public Health Foundation of India, IIPH Hyderabad.<br>Babu, Giridhara R; Public Health Foundation of India; Wellcome Trust DBT India Alliance
**Primary Subject Heading**:	Epidemiology
Secondary Subject Heading:	Epidemiology, Mental health, Nutrition and metabolism, Obstetrics and gynaecology
Keywords:	DIABETES & ENDOCRINOLOGY, Health policy < HEALTH SERVICES ADMINISTRATION & MANAGEMENT, PUBLIC HEALTH, EPIDEMIOLOGY

I, the Submitting Author has the right to grant and does grant on behalf of all authors of the Work (as defined in the below author licence), an exclusive licence and/or a non-exclusive licence for contributions from authors who are: i) UK Crown employees; ii) where BMJ has agreed a CC-BY licence shall apply, and/or iii) in accordance with the terms applicable for US Federal Government officers or employees acting as part of their official duties; on a worldwide, perpetual, irrevocable, royalty-free basis to BMJ Publishing Group Ltd ("BMJ") its licensees and where the relevant Journal is co-owned by BMJ to the co-owners of the Journal, to publish the Work in this journal and any other BMJ products and to exploit all rights, as set out in our licence.

The Submitting Author accepts and understands that any supply made under these terms is made by BMJ to the Submitting Author unless you are acting as an employee on behalf of your employer or a postgraduate student of an affiliated institution which is paying any applicable article publishing charge ("APC") for Open Access articles. Where the Submitting Author wishes to make the Work available on an Open Access basis (and intends to pay the relevant APC), the terms of reuse of such Open Access shall be governed by a Creative Commons licence – details of these licences and which Creative Commons licence will apply to this Work are set out in our licence referred to above.

Other than as permitted in any relevant BMJ Author's Self Archiving Policies, I confirm this Work has not been accepted for publication elsewhere, is not being considered for publication elsewhere and does not duplicate material already published. I confirm all authors consent to publication of this Work and authorise the granting of this licence.

For peer review only

Full Title: Cohort Profile: Maternal antecedents of adiposity and studying the transgenerational role of hyperglycemia and insulin (MAASTHI)

Short title: Cohort profile: MAASTHI

Authors: Eunice Lobo[1], Yamuna Ana[1], R Deepa,[1] Prafulla Shriyan,[1] ND Sindhu,[1] Maithili Karthik,[1] Sanjay Kinra,[2] GVS Murthy,[3,4] Giridhara R. Babu[1,5]

Affiliations:

1. Indian Institute of Public Health-Bengaluru, Public Health Foundation of India (PHFI), Bengaluru, India
2. London School of Hygiene & Tropical Medicine and University College London Hospital, London, United Kingdom
3. International Centre for Eye Health, Department of Clinical Research, London School of Hygiene & Tropical Medicine, London, United Kingdom
4. Indian Institute of Public Health-Hyderabad, Public Health Foundation of India (PHFI), Hyderabad, India
5. Wellcome Trust-DBT India Alliance Intermediate Research Fellow in Public Health, Hyderabad, India

* **Corresponding author**: Dr Giridhara R. Babu,

Address: Indian Institute of Public Health-Bengaluru, Public Health Foundation of India (PHFI), Besides Leprosy Hospital, 1st Cross, Magadi Road, Bengaluru, India - 560023

Email: epigiridhar@gmail.com

For peer review only

Abstract

Purpose: The Maternal Antecedents of Adiposity and Studying the transgenerational role of Hyperglycemia and Insulin (MAASTHI) cohort in Bengaluru, South India, aims to understand the transgenerational role of increased circulating glucose levels or hyperglycemia and other nutrients and psychosocial environment, on the risk of childhood obesity, as an early marker of chronic diseases.

Participants: Through this paper, we describe the baseline characteristics of the cohort participants and their children, along with plans and challenges. A total of 5694 pregnant women were screened, with 4862 (85.4%) eligible pregnant women recruited at baseline. We assessed anthropometry, Haemoglobin status, Oral Glucose Tolerance Test (OGTT), dietary practices, depressive symptoms using the Edinburgh Postnatal Depression Scale (EPDS), and social support in all women. Follow-up visits involved assessing anthropometry and the health profile of mothers and children.

Findings to date: Among the 4862 eligible participants recruited, 3260 (67%) underwent OGTT, while 2962 participants completed OGTT (90.9%). During pregnancy, 9.7% of women were obese (>90th percentile of skinfold thickness), and 14.3% had Gestational Diabetes Mellitus (GDM). Moreover, 6.2% and 16.8% of women had symptoms suggestive of depression during pregnancy and the immediate postnatal period, respectively. We found that 3.3% of children were Small for Gestational Age (SGA), 10.8% were Large for Gestational Age (LGA), and 9.7% of children were obese at birth.

Future plans: We have completed recruitment and baseline data collection in 2019, and are conducting annual follow-ups until age 4 of the participant's children. For delineating causal pathways of childhood obesity, blood aliquots are stored in the biorepository. The study will inform policy formulation and community awareness in the prevention and control of Non-Communicable Diseases (NCDs) and health promotion.

Keywords: *Gestational diabetes, obesity, birth cohort, psychosocial environment, low birth weight, India*

For peer review only

Strengths and limitations of this study

- MAASTHI is a well-established cohort study documenting the life course trajectory in the genesis of NCDs for important exposure contrasts experienced by women very early on in their pregnancy and continues to document the outcomes across the process of pregnancy, childbirth, and early childhood, including assessing important milestones of the child.
- This is the largest prospective birth cohort in the urban public sector on maternal and child health in South India, with a large sample size over a relatively long period of follow-up.
- Blood aliquots are stored in the biorepository for future studies on childhood obesity.
- Operational issues in terms of incomplete OGTT and loss-to-follow-up after delivery are the limitations of conducting a study in public health facilities.
- Our cohort will inform policy formulation and community awareness in the prevention and control of Non-Communicable Diseases (NCDs) and health promotion.

For peer review only

Introduction to MAASTHI cohort

According to the recent global disease burden study, type 2 diabetes mellitus (T2DM) is the fifth commonest disease affecting Indians (1). T2DM is projected to affect 70 million Indians by the year 2025 (2-7) at a relatively younger age compared to high-income countries. The increasing prevalence of Gestational diabetes mellitus (GDM) directly impacts the T2DM burden (6). Findings from the Hyperglycemia and Adverse Pregnancy Outcome (HAPO) study initially confirmed the association between maternal glucose levels and neonatal adiposity (8-11). These results conform to the theoretical frameworks of 'fuel-mediated teratogenesis', 'thrifty phenotype', and thrifty genotype' hypotheses (2, 8, 11-15), which explain the trans-generational nature of obesity-hyperglycemia. Even the Parthenon Birth cohort and others (16-18) have provided initial pieces of evidence regarding this association from low-and-middle-income countries (LMICs). Thus, it becomes necessary to update the recent findings and, more importantly, capture the evidence from resource-constrained, high-burden countries such as India. Given the rapid demographical and epidemiological transition in India, there is a pressing need to capture the evidence on the obesity-hyperglycemia burden (19).

In LMICs, children who have low birth weight (LBW) and early undernutrition are known to have rebound adiposity during early adolescence (20). Therefore, it is important to capture the life course trajectories of the underweight and obesity-led hyperglycemia epidemics in LMICs. The risk factors of LBW may also be modifiable to prevent ensuing obesity epidemics in these countries. Among these risk factors, undernutrition in pregnancy affects nearly 28% of newborns having LBW in South Asia (21, 22). Evidence including high-income countries suggests a putative role in the psychosocial environment and weight of the newborn (23, 24). Additionally, a recent systematic review also showed similar results for low-middle income countries (LMICs), with risk factors including poor social support, history of common mental disorders, etc.(25)

For peer review only - http://bmjopen.bmj.com/site/about/guidelines.xhtml

Individual and household stressors frequent exposure leads to poor mental health, including depression and anxiety (26). The mother's poor mental health throughout pregnancy or after delivery affects mother-child interactions and makes it more difficult for child upbringing (27). The effect of maternal mental health on child growth is likely to be more apparent in low socioeconomic situations due to the relative lack of resources and challenging contextual factors that are associated with poverty.

Aligned with the evidence from the high-income countries, we piloted the feasibility of conducting a multi-centric cohort study in the urban public health facilities of Bengaluru. Informed from the results of the pilot study (28), the principal investigator (GRB) obtained funding to set up a birth cohort titled 'Maternal antecedents of adiposity and studying the transgenerational role of hyperglycemia and insulin' (MAASTHI). The primary objective of the cohort is to investigate the effect of glucose levels in pregnancy on skinfold thickness (adiposity) in infancy as a marker of future obesity and diabetes in offspring, while the secondary objective is to assess the association between the psychosocial environment of mothers and adverse neonatal outcomes including adiposity. By capturing evidence from the mothers and children of low-and-middle-income status visiting health facilities in the public sector, MAASTHI will provide a contextual evidence base resulting in obesity-hyperglycemia in India (9, 28).

For peer review only - http://bmjopen.bmj.com/site/about/guidelines.xhtml

Cohort description

Study design, sample size, study site, and participants

We started the recruitment in the prospective cohort study in April 2016 in the urban public health facilities of Bengaluru, a metropolitan city with a 13 million population in South India. The study protocol with the details of recruitment and methodology is published elsewhere (29). With the assumption of a 5% incidence of childhood obesity at birth in India, a relative risk of 1.5 in the hyperglycaemic group, our estimated sample size with 80% power required to detect a difference at a 95% confidence level was 2936 participants. Considering loss to follow-up by about 60%, we had aimed to recruit 5000 pregnant women. We started recruitment in a 300-bedded public health facility (Jayanagar General Hospital) in Bengaluru corporation. The cohort is established in 14 public health facilities in Bengaluru, Karnataka viz. Jayanagar General Hospital, Bagaluru Referral Hospital, Srirampura Referral Hospital, Anjanappa Garden UPHC, Banashankari Referral Hospital, Bapuji Nagar UPHC, D J Halli UPHC, KC General Hospital, Magadi road UPHC, Pantharapalaya UPHC, Robertsonpet UPHC, Siddaiah Referral Hospital, Sirsi Circle UPHC, and Tavrekere UPHC.

All pregnant women between 14 to 36 weeks of gestation from the ages of 18 to 45 years visiting the public health facilities were approached to participate in the study. After obtaining written informed consent, we recruited the women only if they planned to deliver in the study health facility and were available for future follow-ups in Bengaluru. Exclusion criteria included a history of major co-existing diseases as HIV/ADIS, Hepatitis B, diabetes, and the inability to complete the oral glucose tolerance test (OGTT) within 24 to 36 weeks of gestation.

Patient and public involvement

No patient involvement.

For peer review only

For peer review only - http://bmjopen.bmj.com/site/about/guidelines.xhtml

Data collection

Recruitment and follow-up

We approached 5694 pregnant women and collected the baseline data from 4862 eligible pregnant women and 2873 women who delivered during the period of April 2016 to June 2019. A brief overview of study recruitment, retention, and at-birth follow up, is presented in fig. 1.

After obtaining written informed consent, eligible pregnant women were recruited into the study when they completed 14 weeks of gestational age, and face-to-face interviews were done by trained research assistants ensuring privacy and confidentiality.

What has been measured?

We have collected socio-demographic details, 24-hour dietary recall, dietary habits, use of tobacco and alcohol (own and spouse) and physical activity in the women. In addition to obtaining a family history of diabetes and other cardiovascular diseases (CVD), we obtained an obstetric history from all the pregnant women (Table 1). Additionally, we have assessed the psychosocial environment using validated versions of the Edinburgh Postnatal Depression Scale (EPDS) and social support scale (30). Between 24 to 36 weeks, all the women were invited to undergo OGTT. GDM was classified using the World Health Organisation (WHO) diagnostic criteria (31). We also measured blood pressure, assessed body mass index (BMI), and measured hemoglobin in all the women (Table 1).

The follow-up visits began soon after the delivery. We recorded the health status, morbidities, or any other illness of the baby, feeding and mother's follow-up assessments. The measurements of skinfold thickness, circumferences, and weight, length in infants were taken at birth and 14 weeks, corresponding to the child's immunization visits to the public health facility. Further follow-up visits of the children

For peer review only - http://bmjopen.bmj.com/site/about/guidelines.xhtml

include anthropometric assessment annually at ages 1, 2, 3 and 4; along with other assessments mentioned in Table 1.

Table 1: Assessments and data collected among the participants of MAASTHI birth cohort, Bengaluru 2016

Time	***Variable***
Mother: *14 to 36 weeks*	Socio-demographic details<br>Family medical history<br>Physical activity<br>Tobacco/Alcohol use (Self and spouse)<br>Dietary habits/24-hour recall<br>Obstetrics history<br>Assessment of depressive symptoms (using EPDS) and social support<br>Medications/supplements taken<br>Blood pressure<br>Anthropometry measurements<br>• Weight<br>• Height<br>• Biceps skinfold thickness<br>• Triceps skinfold thickness<br>• Subscapular skinfold thickness<br>• Head circumference<br>• Mid Upper Arm Circumference (MUAC)
24 to 36 weeks	OGTT (Fasting plasma glucose, 2-h postprandial plasma glucose)<br>Haemoglobin
At delivery	Delivery details<br>Mother's weight<br>Cause of death, if any<br>Random Blood Sugar for GDM women<br>Initiation of breastfeeding and feeding practice<br>Assessment of depressive symptoms (using EPDS)
Post-delivery -14 weeks, 1,2,3,4 years	Health status<br>Medications/supplements taken<br>Body Mass Index (BMI)<br>Blood pressure<br>Assessment of depressive symptoms (using EPDS)<br>Social support<br>Anthropometry measurements<br>• Weight<br>• Waist circumference<br>• Hip circumference<br>• Biceps skinfold<br>• Triceps skinfold<br>• Subscapular skinfold
Child*: At birth, 14th week, 1,2,3, 4 years*	Body Mass Index (BMI)<br>Anthropometry measurements<br>• Weight<br>• Length<br>• Crown-rump length (birth to 1 year)<br>• Head circumference

For peer review only - http://bmjopen.bmj.com/site/about/guidelines.xhtml

	• Chest circumference<br>• Waist circumference<br>• Hip circumference<br>• Mid upper arm circumference<br>• Biceps skinfold thickness<br>• Triceps skinfold thickness<br>• Subscapular skinfold thickness<br>Infant data & neonatal problems<br>Morbidity/disease/Neonatal Intensive Care Unit/Paediatric Intensive Care Unit admissions<br>Immunization records<br>Medications & supplement use<br>Diet<br>Milk or formula feeding<br>Introduction of complementary food and reason<br>Cessation of breastfeeding and reason
14 weeks, 1,2,3, 4 years	Trivandrum Developmental Screening Chart
2 years	Toddler feeding practice<br>Modified Checklist for Autism in Toddlers<br>Physical activity

Fasting plasma glucose equal to or more than 92 mg/dL or 2-h postprandial plasma glucose equal to or more than 152 mg/dL(32) as GDM.

Abbreviation: OGTT: Oral Glucose Tolerance Test; EPDS: Edinburgh Postnatal Depression Scale

We use the support from frontline health workers in the community to remind and help track the unavailable participants for follow-up visits. Additionally, the research staff regularly call the participants for reminders regarding the follow-up schedule, and if required, also do house visits (after prior permission). These additional efforts are done for participants who cannot visit the public health facility during the defined period. To make it easier for participants, we also schedule follow-up visits in the primary health centres and *Anganwadi* centres near their residence. We have also examined the role of an interactive voice-based response system (IVRS) and an information workshop in improving the follow-up rates in these women (32).

Through IVRS, women received a 2–3 minutes call to inform them about OGTT test dates, collect their laboratory test reports, and remind them of their follow-up visits. The IVRS also provided health information on Gestational Diabetes Mellitus (GDM), breastfeeding, and immunization.

A Mother and Baby Affairs (MBA) workshop or antenatal workshop and counselling for parents were conducted. This included a lunch for the participant and her family (husband, and children, if any). Generally, this is called "*Seemantha*" in *Kannada*, similar to a baby shower in other parts of the world.

For peer review only - http://bmjopen.bmj.com/site/about/guidelines.xhtml

The workshop included a brief talk by health professionals on the importance of GDM screening and management and other antenatal and postnatal topics.

Storage of blood samples for future analysis

At 24 to 36 weeks, during the collection of blood for OGTT, we collect 11 ml venous blood samples in the fasting state and 2 ml for postprandial blood glucose analysis. The blood is collected in three vacutainers; plain (6 ml for storage), Ethylenediaminetetraacetic acid (EDTA) (3 ml for Hemoglobin analysis), and sodium fluoride vacutainers (2 ml each for glucose analysis in fasting and postprandial), respectively. Blood samples are centrifuged and transferred to cryovials within an hour of collecting in cool boxes to a single central laboratory for assays with external quality assurance mechanisms.

Measurements

We perform anthropometric measurements of mothers at the baseline and during subsequent follow-up visits. The weight was measured using Tanita weighing scale, and height using the SECA 213 portable stadiometer. The mother's blood pressure (BP) is measured using the automated digital device (Omron Digital BP measuring device). The anthropometric measurements of the baby are recorded within 72 hours of delivery. Newborn anthropometry was performed using SECA 354 Weighing Scale and SECA 417 Infantometer. We measure the head, mid-upper arm (MUAC), chest, and waist circumferences in both the mother and child using Chasmors body circumference tape. The sum of skinfold thickness (biceps, triceps, and subscapular) (SFT) is measured on the left side of the body using the Holtain Calipers (Holtain Ltd, UK).

review only

Quality control

For peer review only - http://bmjopen.bmj.com/site/about/guidelines.xhtml

Research assistants were trained and certified by St. Johns Research Institute, Bengaluru, in anthropometric measurements. Strict protocols are followed to maintain accuracy, with the addition of inter-and intra-observer reliability of measurements assessed at the outset followed by annual certification by the same institute. Trained phlebotomists collect venous blood for laboratory investigations. Biochemical assays are conducted at a central nationally-accredited laboratory with internal and external quality checks. Calibration of all the equipments is done every month using prescribed guidelines, with a calibration log maintained by the research staff and supervised by the principal investigator of the study.

Data management and analysis

Data is entered on android tablets regularly using the application specifically designed for the study. The details of the development and use are published elsewhere (33). The primary data collected is exported through MS Excel 2010 and checked periodically by senior team members for data entry errors and missing information. For this paper, the exported data was then cleaned, and explorative data analysis was done to understand and summarize the variables involved. The descriptive analysis was performed using SPSS version 23. The modified EPDS scores cut-off for defining symptoms suggestive of depression was 13 based on a previous study in Karnataka(34), and social support scores were categorized based on the cut-off score of 24 and above as good(≥24) and poor(<24)(35). Adiposity (mother and child) and gestational age (child) weight were classified based on percentiles - using 90th percentile as the cut-off value. Adiposity was considered based on the total sum of skinfold thickness (using biceps, triceps and subscapular thickness). At the same time, weight for gestational age was classified considering the weight of children at each gestational age, adjusted for parity and gender(36). The birth weight of children was categorized based on the WHO standards, and preterm births were defined as gestational age less than 37 weeks (37). Anaemia in pregnant women was categorized on the WHO criteria based on haemoglobin

For peer review only - http://bmjopen.bmj.com/site/about/guidelines.xhtml

concentration: Anemic when <110 g/L at sea level, and no anemia when above 100g/L. (38) Hypertension among participants was categorized into: Normal (<120/80 mmHg), Pre-hypertension (120-139/80-89 mmHg), Stage 1 hypertension (140-159/90-99 mmHg), and Stage 2 hypertension (≥160/100 mmHg)(39). Body mass index (BMI) of the participants was categorized based on the WHO Asian criteria: Underweight (<18.5), Normal (18.5-22.9), Overweight (23.0-24.9), and Obese(>25.0)(40). Socioeconomic status was categorized using the modified *Kuppuswamy* socioeconomic status scale 2017 that classifies socioeconomic status as: lower, middle, and upper classes(41).

For peer review only

For peer review only - http://bmjopen.bmj.com/site/about/guidelines.xhtml

Findings to date and discussion

Among 5694 pregnant women, 85.3% (n=4862) were included in the study based on the eligibility criteria and willingness to participate. A total of 67% of women underwent OGTT (n=3260) as per the schedule between 24 to 36 weeks, with a completion rate of 90.9% (n=2962) (Fig. 1). At the baseline, the mean age of the pregnant women was 24.25 ±4.06 years, with 66% between the age group of 18 to 25 years. Most pregnant women had high school education (66%; n=437). More than 90% of women were homemakers (90.0%; n=4446), while most of their spouses were unskilled workers (46.7%; n=2237%). Using the modified *Kuppuswamy* scale, most participants were categorized as lower socioeconomic groups (54.6%; n= 2614). (Table 2)

We found the prevalence of Gestational diabetes mellitus in these women was 14.3% with 95% Confidence Interval (CI): 0.13-0.16 (n=424/2962). Among the recruited participants, 265 were pre-hypertensive (7.1%; 95% CI:0.69 - 0.72), and 15 were hypertensive (0.4%; 95% CI: 0.0020 - 0.0060), while 44.5% of the women were anemic (n=1377; 95% CI: 0.42 - 0.46). Using the cut off value of 13 for EPDS, we found that 6.2% of women (n=289) had depressive symptoms indicative of depression during pregnancy, and symptoms of postnatal depression were present in 16.8% of women (n=471). The mean gestational age at delivery was 38.7 ± 3.02 weeks. More than half of the newborns were males (51.9%; n=1717), 43.8% of the infants were delivered by caesarean section (n=1456), and 91.2% infants were born full-term (n=4432). (Table 2)

For peer review only - http://bmjopen.bmj.com/site/about/guidelines.xhtml

Table 2: Baseline characteristics of eligible pregnant mothers and children in the MAASTHI birth cohort, Bengaluru, 2016-2021

**Maternal characteristics**	
**Socio-demographic characteristics**	**n (%)**
**Age (years) (N=4862)**	
Mean age± SD	24.25 ± 4.06
18-25	3211 (66.0)
26-35	1598 (32.9)
36-45	53 (1.1)
**Religion (N=4862)**	
Hindu	2454 (50.5)
Muslim	2142 (44.1)
Others	266 (5.5)
**Consanguineous marriage (N=4788)**	1157 (24.2)
**Participant's education (N=4788)**	
Illiterate	169 (3.5)
Primary school	284 (5.9)
Middle school	609 (12.7)
High school	2091 (43.7)
PUC or diploma	1072 (22.4)
Graduate and above	563 (11.8)
**Husband's education (N=4788)**	
Illiterate	426 (8.9)
Primary school	461 (9.6)
Middle school	724 (15.1)
High school	1956 (40.9)
PUC or diploma	793 (16.6)
Graduate and above	422 (8.8)
Do not know	6 (0.1)
**Participant's occupation (N=4788)**	
Homemaker	4446 (92.9)
Unskilled worker	168 (3.5)
Semiskilled worker	76 (1.6)
Skilled worker	85 (1.8)
Professional	13 (0.3)
**Husband's occupation (N=4788)**	
Unemployed	17 (0.4)
Unskilled worker	2237 (46.7)
Semiskilled worker	1535 (32.1)
Skilled worker	860 (18.0)
Professional	139 (2.9)
**Socio-economic status (N=4788)**	
Lower class	2614 (54.6)
Middle class	2145 (44.8)
Upper class	29 (0.6)
**Chronic conditions with 95% CI of prevalence estimates**	
**Hypertension (N=3719)**	

For peer review only

For peer review only - http://bmjopen.bmj.com/site/about/guidelines.xhtml

Normal<br>Pre hypertension<br>Stage 1 hypertension	3429 (92.2) (95% CI: 0.91 - 0.93)<br>265 (7.1) (95% CI: 0.69 - 0.72)<br>15 (0.4) (95% CI: 0.0020 - 0.0060)
**GDM during current pregnancy (N=2962)**	424 (14.3) (95% CI: 0.13 - 0.16)
**Anemia (N=3097)**	1377 (44.5) (95% CI: 0.42 - 0.46)
**Obstetrical characteristics (N=4778)**	
**Gravida**	
Primigravida<br>Multigravida	1920 (40.2)<br>2858 (59.8)
**Parity**	
Nulliparous<br>Multiparous	2178 (45.6)<br>2600 (54.4)
**Mean Gestational age at delivery (week) (N=3324)**	38.70 ± 3.02
**Depressive symptoms and social support**	
EPDS score at pregnancy (>13 cut-off) (N=4635)<br>Social support score above 24 (N=4643)<br>EPDS score at delivery (>13 cut-off) **(N=2801)**	289 (6.2)<br>1520 (32.7)<br>471 (16.8)
**Health status of mothers at delivery (N=3324)**	
Healthy<br>Morbidity/Hospitalization *Death	3054 (92.0)<br>259 (7.8)<br>7 (0.2)
**Child characteristics n (%)**	
**Health status at birth (N=3310)**	
Healthy<br>Morbidity/Hospitalization**<br>Death	3188 (96.3)<br>49 (1.5)<br>73 (2.2)
**Preterm deliveries (N=4862)**	430 (8.8)
**Birth weight (N=4862)**	
Underweight<2500g<br>Normal 2500 g to 3500 g<br>Overweight >3500g	384 (7.9)<br>1625 (33.4)<br>2853 (58.7)
**Birth weight according to Gestational Age (N=4862)**	
Small for Gestational Age (SGA)<br>Mean ± SD weight for SGA (kg)<br>Large for Gestational Age (LGA)<br>Mean ± SD weight for LGA (kg)	159 (3.3)<br>2.13 ± 0.26<br>523 (10.8)<br>4.10 ± 0.72
**Delivery type (N=3324)**	
Vaginal delivery<br>Caesarean section	1868 (56.2)<br>1456 (43.8)
**Sex (N=3309)**	
Male<br>Female	1717 (51.9)<br>1592 (48.1)
**Baby cried soon after delivery (N=3310)**	3030 (91.5)
**Colostrum fed to the baby (N=2915)**	2562 (87.9)
**Breast milk fed as first food to the baby (N=2915)**	1932 (66.3)
**Initiation of breast feeding after delivery (N=2890)**	
Within an hour<br>Between 1-24 hour<br>After 24 hours	1227 (42.4)<br>1538 (53.2)<br>125 (4.4)

For peer review only

For peer review only - http://bmjopen.bmj.com/site/about/guidelines.xhtml

Abbreviation: EPDS: Edinburgh Postnatal Depression Scale

GDM: Fasting blood sugar (FBS) equal to or more than 92 mg/dL or 2-h postprandial blood sugar (PPBS) equal to or more than 152 mg/dL

Hypertension: Normal: SBP <120mmHg and DBP <80mmHg; prehypertension: SBP 120-139mmHg or DBP 80-89mmHg; Stage 1 hypertension: SBP 140-159mmHg or DBP 90-99mmHg

*Morbidity/Hospitalization (mother): postpartum hemorrhage, anaemia, infections, hypertensive disorder, diabetes mellitus, respiratory illness, thyroid disease

**Morbidity/ Hospitalization (newborn): fever, admission to NICU due to jaundice, low birth weight, IUGR, allergy, asphyxia, respiratory distress syndrome, Gastrointestinal abnormalities, congenital anomaly, macrocephaly, birth trauma, meconium aspiration, premature baby, infections, convulsions, pneumonia, hypoglycemia

Table 3 and 4 summarizes the anthropometric measurements of the participants (mothers) and their offspring during each phase of the study period respectively. Categorization is based on tertiles -10th, 10 – 90th, and >90th percentiles. Each table includes mean and standard deviation (SD) values for the measurements described in the analysis, and the frequency and proportion of mother and child in each tertile. We found that among the mothers above the 90th percentile for weight, 9.9% were obese at delivery and subsequent follow-ups. They weighed an average of 82.74 kg as compared to 83.84 kg at the 3-years follow-up. During the period from delivery to the 3-year follow up, the average SFT in women with obesity (>90th percentile of SFT) changed from 76.6 mm (9.7%) to 75.46 mm (9.9%) in comparison to women with a normal range of SFT (10 to 90th percentile) in whom it changed from 46.54 mm (80.6%) to 49.79 mm (80.2%) (Table 3).

For peer review only

For peer review only - http://bmjopen.bmj.com/site/about/guidelines.xhtml

Table 3: Anthropometry of the mother at different follow-up periods of the MAASTHI birth cohort, Bengaluru, 2016-21

**Anthropometric measurement**		**During Pregnancy (N=3734)**		**14th week follow up (N=2146)**		**1 year follow up (N=1255)**		**2 year follow up (N=801)**		**3 year follow up (N=393)**	
		**n (%)**	**Mean (SD)**	**n (%)**	**Mean (SD)**	**n (%)**	**Mean (SD)**	**n (%)**	**Mean (SD)**	**n (%)**	**Mean (SD)**
Weight	<10th percentile	367 (9.8)	41.88 (2.54)	214 (10.0)	40.35 (2.73)	124 (9.9)	38.96 (2.21)	80 (10.0)	40.25 (2.91)	39 (9.9)	40.41 (2.04)
	10-90th percentile	2996 (80.2)	58.09 (7.60)	1720 (80.1)	56.90 (7.76)	1006 (80.2)	56.54 (8.00)	642 (80.1)	59.25 (8.18)	315 (80.2)	59.27 (8.31)
	>90th percentile	371 (9.9)	82.74 (7.07)	212 (9.9)	81.27 (6.56)	124 (9.9)	81.79 (7.02)	79 (9.9)	85.86 (8.51)	39 (9.9)	83.84 (7.07)
Waist circumference	<10th percentile	-	-	194 (9.6)	61.51 (3.71)	124 (9.9)	60.18 (2.97)	80 (10.0)	61.04 (2.71)	38 (9.7)	60.27 (3.65)
	10-90th percentile	-	-	1635 (80.8)	77.96 (7.51)	1006 (80.2)	77.43 (7.61)	641 (80.0)	79.87 (8.41)	319 (81.2)	79.01 (8.06)
	>90th percentile	-	-	195 (9.6)	99.46 (6.23)	125 (10)	98.99 (6.15)	80 (10.0)	103.59 (6.00)	36 (9.2)	98.82 (4.07)
MUAC	<10th percentile	358 (9.6)	20.01 (1.15)	190 (8.8)	20.57 (1.02)	124 (9.9)	20.39 (1.19)	74 (9.2)	20.59 (1.44)	39 (9.9)	20.44 (1.41)
	10-90th percentile	3002 (80.4)	25.78 (2.55)	1744 (81.2)	26.42 (2.52)	1012 (80.6)	26.74 (2.62)	649 (81.0)	27.65 (2.68)	315 (80.2)	27.30 (2.65)
	>90th percentile	372 (10.0)	33.54 (2.11)	213 (9.9)	34.03 (2.08)	119 (9.5)	43.43 (2.05)	78 (9.7)	35.30 (2.05)	39 (9.9)	34.44 (1.66)
Total SFT	<10th percentile	361 (9.7)	25.21 (2.91)	213 (9.9)	24.72 (3.51)	124 (9.9)	22.44 (3.38)	79 (9.9)	25.37 (4.19)	39 (9.9)	24.19 (4.12)

For peer review only - http://bmjopen.bmj.com/site/about/guidelines.xhtml

	10-90th percentile	3008 (80.6)	46.54 (10.05)	1723 (80.3)	46.84 (9.59)	1007 (80.2)	45.93 (10.17)	641 (80.1)	50.69 (10.20)	315 (80.2)	49.79 (10.17)
	>90th percentile	363 (9.7)	76.60 (8.31)	210 (9.8)	73.83 (6.75)	125 (10.0)	73.92 (6.04)	80 (10.0)	79.55 (8.15)	39 (9.9)	75.46 (7.00)
Hip Circumference	<10th percentile	-	-	199 (9.8)	79.39 (4.85)	121 (9.6)	77.52 (4.00)	80 (10.0)	78.71 (5.93)	39 (9.9)	80.33 (3.29)
	10-90th percentile	-	-	1624 (80.2)	94.99 (6.71)	1013 (80.7)	94.75 (7.06)	642 (80.1)	97.18 (7.16)	315 (80.2)	96.69 (6.89)
	>90th percentile	-	-	201 (9.9)	115.35 (5.47)	121 (9.6)	115.47 (5.71)	79 (9.9)	118.56 (5.75)	39 (9.9)	115.63 (3.29)
Height	<10th percentile	368 (9.9)	144.15 (2.27)	-	-	-	-	-	-	-	-
	10-90th percentile	2993 (80.2)	153.81 (3.80)	-	-	-	-	-	-	-	-
	>90th percentile	373 (10.0)	164.16 (2.77)	-	-	-	-	-	-	-	-
Head circumference	<10th percentile	329 (8.8)	49.46 (1.09)	-	-	-	-	-	-	-	-
	10-90th percentile	3080 (82.5)	52.69 (1.15)	-	-	-	-	-	-	-	-
	>90th percentile	324 (8.7)	56.08 (1.00)	-	-	-	-	-	-	-	-

Anthropometric measurements of children showed that the sum of skinfold thickness ranged from 22.37 mm to 32.6 mm from birth to the 3-year follow-up for children above the 90th percentile compared to 13.76 mm to 22.87 mm, respectively in children between 10 to the 90th percentile. Birth of a high

For peer review only - http://bmjopen.bmj.com/site/about/guidelines.xhtml

proportion of obese children (weight above 90th percentile), i.e., 9.7%, was noted in our study (Table 4). We also noted that 7.9% of children born were low birth weight (2500 gms or less), and 8.8% (n=430) were born preterm. A high proportion of 10.8% (n=523) of children born were large for gestational age (LGA) in our cohort, and 3.3% were small for gestational age (SGA) (n=159). The mean weight of children who were SGA at birth was 2.13 ± 0.26 kg and was 4.10 ± 0.72 kg for children with LGA.

For peer review only

Table 4: Anthropometry of the child at different follow-up periods of the MAASTHI birth cohort, Bengaluru, 2016-21

Anthropometric measurement		At birth		14 week follow up		1 year follow up		2 year follow up		3 year follow up	
		n (%)	Mean (SD)	n (%)	Mean (SD)	n (%)	Mean (SD)	n (%)	Mean (SD)	n (%)	Mean (SD)
Weight	<10th percentile	243 (9.8)	2.14 (0.21)	182 (8.5)	4.31 (0.39)	122 (9.7)	6.84 (0.38)	74 (9.0)	8.56 (0.62)	41 (10.0)	9.80 (0.64)
	10-90th percentile	2003 (80.5)	2.98 (0.41)	1761 (82.4)	6.04 (0.72)	1010 (80.5)	8.74 (0.82)	670 (81.5)	10.73 (0.90)	333 (81.2)	12.30 (0.95)
	>90th percentile	242 (9.7)	4.78 (0.74)	195 (9.1)	8.37 (0.73)	122 (9.7)	11.43 (0.85)	78 (9.5)	13.41 (0.77)	36 (8.8)	14.74 (0.28)
CRL	<10th percentile	233 (9.4)	27.37 (2.01)	213 (10.0)	33.21 (1.89)	114 (9.9)	37.28 (2.48)	-	-	-	-
	10-90th percentile	2020 (81.5)	32.41 (2.01)	1719 (80.6)	40.52 (2.40)	926 (80.2)	45.31 (2.36)	-	-	-	-
	>90th percentile	226 (9.1)	39.29 (2.21)	202 (9.5)	47.66 (2.79)	114 (9.9)	53.44 (4.00)	-	-	-	-
Length	<10th percentile	248 (10.0)	43.69 (1.72)	206 (9.6)	55.26 (2.22)	125 (10.0)	65.76 (6.28)	79 (9.6)	74.64 (3.09)	41 (10.0)	83.16 (3.42)
	10-90th percentile	2009 (80.8)	49.38 (2.28)	1720 (80.5)	62.66 (2.74)	1003 (80.2)	73.79 (2.65)	664 (80.8)	83.17 (2.92)	329 (80.2)	90.78 (2.77)
	>90th percentile	230 (9.2)	58.06 (2.81)	211 (9.9)	70.99 (2.24)	123 (9.8)	81.68 (2.78)	79 (9.6)	91.61 (2.23)	40 (9.8)	97.89 (1.65)
Head Circumference	<10th percentile	242 (9.7)	30.30 (1.07)	199 (9.3)	36.30 (2.05)	102 (8.1)	40.39 (1.79)	81 (9.9)	42.49 (1.45)	39 (9.5)	43.96 (0.87)
	10-90th percentile	2001 (80.4)	33.66 (1.33)	1740 (81.4)	40.07 (1.33)	1033 (82.4)	44.23 (1.16)	666 (81.3)	46.16 (1.25)	332 (81.0)	47.10 (1.09)
	>90th percentile	245 (9.8)	38.10 (1.25)	199 (9.3)	43.97 (0.96)	119 (9.5)	47.41 (1.33)	72 (8.8)	50.81 (4.86)	39 (9.5)	50.27 (1.48)
Chest circumference	<10th percentile	246 (9.9)	28.10 (1.19)	198 (9.3)	35.67 (1.02)	125 (10.0)	39.73 (1.89)	69 (8.4)	42.54 (1.41)	38 (9.3)	44.57 (0.87)
	10-90 percentile	2014 (80.9)	32.24 (1.73)	1740 (81.4)	40.12 (1.71)	1017 (81.1)	44.35 (1.58)	673 (81.8)	46.78 (1.58)	333 (81.2)	48.56 (1.69)
	>90th percentile	228 (9.2)	38.38 (1.83)	200 (9.4)	45.08 (1.27)	112 (8.9)	49.15 (1.48)	81 (9.8)	56.49 (1.64)	39 (9.5)	53.0 (1.04)
Waist circumference	<10th percentile	235 (9.4)	24.81 (1.17)	182 (8.5)	33.14 (1.46)	2 (0.2)	29.30 (1.69)	77 (9.3)	40.17 (1.64)	41 (10.0)	41.93 (1.20)
	10-90 percentile	2008 (80.7)	30.66 (2.47)	1747 (81.7)	39.28 (2.29)	1132 (90.3)	41.95 (2.86)	667 (80.9)	45.57 (1.98)	329 (80.2)	46.94 (2.09)
	>90th percentile	245 (9.8)	38.24 (1.87)	209 (9.8)	45.37 (1.58)	119 (9.5)	49.02 (1.83)	80 (9.7)	51.39 (1.96)	40 (9.8)	53.27 (1.40)
Hip Circumference	<10th percentile	211 (8.5)	23.67 (0.93)	211 (9.9)	32.05 (1.49)	4 (0.3)	30.75 (2.12)	79 (9.6)	39.71 (1.47)	39 (9.5)	41.70 (4.69)
	10-90 percentile	2037 (81.9)	28.91 (2.44)	1717 (80.3)	39.05 (2.85)	1125 (89.7)	42.17 (3.06)	669 (81.2)	46.23 (2.51)	332 (81.0)	48.71 (2.50)

For peer review only

	>90th percentile	240 (9.6)	37.32 (2.68)	210 (9.8)	46.42 (1.60)	125 (10.0)	50.0 (1.97)	76 (9.2)	53.47 (1.87)	39 (9.5)	55.21 (1.31)
MUAC	<10th percentile	191 (7.7)	7.86 (0.58)	206 (9.6)	10.58 (0.76)	124 (9.9)	11.25 (0.96)	78 (9.5)	11.65 (0.82)	39 (9.5)	12.28 (1.47)
	10-90 percentile	2074 (83.4)	9.81 (0.76)	1748 (81.8)	12.88 (0.82)	1015 (80.9)	13.77 (0.84)	665 (80.8)	14.15 (0.80)	333 (81.2)	14.57 (0.73)
	>90th percentile	223 (9.0)	12.48 (0.77)	183 (8.6)	15.31 (0.70)	115 (9.2)	16.25 (1.52)	80 (9.7)	16.72 (1.03)	38 (9.3)	17.76 (5.78)
Total SFT	<10th percentile	223 (9.0)	9.27 (0.78)	196 (9.2)	14.25 (1.20)	119 (9.5)	14.36 (0.98)	78 (9.5)	14.94 (1.20)	40 (9.8)	15.53 (1.06)
	10-90 percentile	2005 (81.0)	13.76 (2.14)	1723 (81.2)	20.80 (2.61)	1006 (80.5)	19.98 (2.47)	670 (81.4)	21.58 (3.05)	332 (81.0)	22.87 (3.41)
	>90th percentile	246 (9.9)	22.37 (3.25)	204 (9.6)	29.45 (3.31)	124 (9.9)	28.02 (2.74)	75 (9.1)	32.46 (3.74)	38 (9.3)	32.60 (3.28)

Strengths and limitations

As there is no unanimity regarding appropriate guidelines for diagnosing GDM in the Indian population, our findings can provide specific evidence regarding exact cut-off points to be used for the diagnosis. The high prevalence of GDM may provide evidence for a policy decision to scale up screening and management of GDM in all the public health facilities. Above this it may be associated with adverse maternal and childhood outcomes. Also, the huge burden of depressive symptoms among mothers, draws attention to the importance of mental health screening as part of routine antenatal care.

We found a high number of children born LGA. Additionally, the prevalence of obesity at birth and subsequent follow-ups was also high (above 9%). If unchanged modifiable factors of obesity such as diet, exercise, and GDM, will continue the vicious cycle of afflicting subsequent generations (42). The results from our cohort will continue to inform and shape policies regarding screening, appropriate care, and management for persons affected with GDM, perinatal depression, and childhood obesity.

MAASTHI is a well-established cohort study documenting the life course trajectory in the genesis of NCDs. We are documenting important exposure contrasts experienced by women early in their pregnancy and continue to document the outcomes across the process of pregnancy, childbirth and early childhood,

For peer review only

including assessing important milestones of the child. In India, public health facilities are the preferred choice for many women (43)from the lower social strata. Our findings revealed an unusually high burden of GDM and maternal obesity similar to earlier studies (44-47) for women between the age group of 18 to 45 years. Our cohort study will inform policies in achieving further reductions in maternal and fetal mortality and morbidity. For example, achieving the desired level of glycemic control during pregnancy can reduce several maternal and fetal adverse effects (48). Early screening and timely management of GDM may prevent some poor outcomes for mother and child.

The strength of MAASTHI is that data pertains to the pregnant women and children belonging to lower- and middle-income socioeconomic status-seeking healthcare from the public health facilities with a strong representation of minority groups and vulnerable sections of the society. Also, this is probably the only pregnancy cohort recruited from public health facilities in India. Although the acronym suggests the transgenerational role of glucose and insulin, the study was conceptualized to include important maternal determinants of underweight and childhood obesity. The comprehensive exposure assessments in MAASTHI include the role of nutritional, psychosocial, and, recently more relevant - air pollution throughout pregnancy and different time points of the life course (49). Attention to obtaining high-quality data by maintaining stringent quality checks for reproducible measurements and minimal errors is another major strength of this study. Moreover, blood aliquots are stored in the biorepository for future studies on childhood obesity.

Operational issues in terms of incomplete OGTT and loss-to-follow-up after delivery are the limitations of conducting a study in public health facilities. Without a registry or defined population for health care services, this limitation will continue to affect implementing cohort studies in public health facilities. Periods of prolonged fasting (previous night) coupled with the issue of gastritis/morning sickness in pregnant women are other factors responsible for incomplete OGTT. As with most research during COVID-

For peer review only - http://bmjopen.bmj.com/site/about/guidelines.xhtml

19, our cohort was also subject to data collection issues. Our research staff had to resolve the inability of physical contact for face-to-face interviews through telephonic interviews. However, no physical meetings meant the complete stop to anthropometric measurements for mother-child dyads.

By setting up and following up this cohort, we can inform the design and implement interventions focusing on health promotion and disease prevention, address undernutrition and overweight epidemics, and thereby contribute to reducing the NCD disease profile in the country.

For peer review only

For peer review only - http://bmjopen.bmj.com/site/about/guidelines.xhtml

Collaboration

The MAASTHI prospective cohort will continue to collect data on various exposures and maternal and child health related outcomes. For more information, refer to the website: maasthi.com. MAASTHI encourages proposals from outside investigators: Proposals involving the use of existing data or the collection of new data. Researchers interested in collaboration are invited to submit requests for MAASTHI clinical data through a research proposal to giridhar@phfi.org

For peer review only

Abbreviations

ANC: Ante-natal check-up; BBMP: Bruhat Bengaluru Mahanagara Palike; BMI: Body Mass Index; EDTA: Ethylene Diamine Tetra Acetic acid; CC: Chest circumference; CRL: Crown rump length; CRP: C Reactive Protein, EPDS: Edinburgh Postnatal Depression Scale; GDM: Gestational diabetes mellitus; HAPO: Hyperglycemia and adverse pregnancy outcome study; HC: Head circumference; HIV: Human immunodeficiency virus; IEC: Institutional Ethics Committee; IIPH: Indian Institute of Public Health; IL: interleukin; LMIC: Low- and middle-income countries; LGA: Large for gestational age; LBW: Low birth weight; MAASTHI: Maternal antecedents of adiposity studying the transgenerational role of hyperglycaemia and insulin; MBA: Mother and baby affairs; MCHAT: Modified Checklist for Autism in Toddlers; MUAC: Middle-upper arm circumference; NCD: Non-Communicable Disease; OGTT: Oral glucose tolerance test; SD: Standard Deviation; SFT: Sum of skinfold thickness; SGA: Small for gestational age; T2DM: Type 2 diabetes mellitus; TNF: tumor necrosis factor ; WC: Waist circumference; WHO: World Health Organization

For peer review only

For peer review only - http://bmjopen.bmj.com/site/about/guidelines.xhtml

Acknowledgements

We sincerely thank the Department of Health and Family Welfare, the Government of Karnataka (DoHFW, GoK) and the Bruhat Bengaluru Mahanagara Palike (BBMP) for permitting us to conduct the study and providing constant support. We thank hospitals under DoHFW, GoK, Superintendents, Medical Officers, Doctors, and all the department support staff for supporting an ongoing study. We thank Dr Sumathi Swaminathan and her team at St John's Research Institute for training the Research Assistants in anthropometry measurement. Our sincere thanks to the Welcome Trust, DBT India Alliance, for the support with funding and guidance. Our sincere thanks to Dr. Suresh Shapeti and Mr. T.S. Ramesh for facilitating the administrative support and coordination. We thank all the research team members of MAASTHI for their support in carrying out research activities in the field. We thank all participants for their effort to enroll and continuous participation in the ongoing cohort.

Funding

This work was supported by the Wellcome Trust/DBT India Alliance Senior Fellowship [Grant No. IA/CPHS/20/1/505278] awarded to Giridhara R. Babu. The funding agency had no role in the design and conduct of the study, collection, management, analysis, and interpretation of the data, preparation, review, or approval of the manuscript, or decision to submit the manuscript for publication.

Conflict of interest

The authors declare that they have no conflict of interest.

For peer review only

For peer review only - http://bmjopen.bmj.com/site/about/guidelines.xhtml

Ethics consideration

The study was reviewed and approved by the Institutional ethics committee (IEC) of the Indian Institute of Public Health – Bengaluru (Approval number IIPHHB/TRCIEC/091/2015 dated 13th November 2015). The progress of the study is reviewed by the IEC, and permissions are extended annually thereafter. Informed consent prior to participation in the study was obtained from all voluntarily willing participants. The written informed consent is obtained for participation, follow-up, and permission to publish anonymous data in any report, journal and for assessing the stored samples for study-related hypotheses. The study was conducted in accordance with the Declaration of Helsinki.

Authors contributions

GRB was involved in the conception, design, and funding acquisition. YA, DR, PS, MK, SN performed the experiments. EL, GRB were involved in drafting the paper and revising the draft. SK, GVSM were involved in the critical analysis of the paper. All the authors have approved the final version of the article submitted.

Data Sharing Statement

To discuss our data sharing policy, please contact Giridhara R. Babu at giridhar@phfi.org

For peer review only

For peer review only - http://bmjopen.bmj.com/site/about/guidelines.xhtml

References

1. India : Health of the Nation's States - The India State-level Disease Burden Initiative. 2017.
2. Hales CN, Barker DJ. The thrifty phenotype hypothesis. British medical bulletin. 2001;60:5-20.
3. Mohan V, Sandeep S, Deepa R, Shah B, Varghese C. Epidemiology of type 2 diabetes: Indian scenario. The Indian journal of medical research. 2007;125:217-30.
4. Shaw JE, Sicree RA, Zimmet PZ. Global estimates of the prevalence of diabetes for 2010 and 2030. Diabetes research and clinical practice. 2010;87:4-14.
5. Anjana RM, Pradeepa R, Deepa M, Datta M, Sudha V, Unnikrishnan R, et al. Prevalence of diabetes and prediabetes (impaired fasting glucose and/or impaired glucose tolerance) in urban and rural India: Phase I results of the Indian Council of Medical Research–INdia DIABetes (ICMR–INDIAB) study. Diabetologia. 2011;54:3022-7.
6. Mithal A, Bansal B, Kalra S. Editorial Gestational diabetes in India : Science and society. 2015:155-8.
7. Prasad A. Type 2 diabetes mellitus in young: need for early screening. Indian pediatrics. 2011;48(9):683.
8. Buchanan T, Freinkel N, Lewis NJ, Metzger BE, Akazawa S. Fuel-mediated teratogenesis. Use of D-mannose to modify organogenesis in the rat embryo in vivo. Journal of Clinical Investigation. 1985;75:1927-34.
9. Group THSCR. Hyperglycemia and Adverse Pregnancy Outcomes. New England Journal of Medicine. 2008;358:1991-2002.
10. HAPO Study Cooperative Research Group THSCR. Hyperglycemia and Adverse Pregnancy Outcome (HAPO) Study: associations with neonatal anthropometrics. Diabetes. 2009;58:453-9.
11. Yajnik CS. Nutrient-mediated teratogenesis and fuel-mediated teratogenesis: two pathways of intrauterine programming of diabetes. International journal of gynaecology and obstetrics: the official organ of the International Federation of Gynaecology and Obstetrics. 2009;104 Suppl 1:S27-31.
12. Neel JV. Diabetes mellitus: a "thrifty" genotype rendered detrimental by "progress"? American journal of human genetics. 1962;14:353-62.
13. Freinkel N. Banting Lecture 1980. Of pregnancy and progeny. Diabetes. 1980;29:1023-35.
14. Hattersley AT, Tooke JE. The fetal insulin hypothesis: an alternative explanation of the association of low bir thweight with diabetes and vascular disease. The Lancet. 1999;353:1789-92.
15. Fetita L-S, Sobngwi E, Serradas P, Calvo F, Gautier J-F. Consequences of Fetal Exposure to Maternal Diabetes in Offspring. The Journal of Clinical Endocrinology & Metabolism. 2006;91:3718-24.
16. Fall CHD, Stein CE, Kumaran K, Cox V, Osmond C, Barker DJP, et al. Size at birth, maternal weight, and Type 2 diabetes in South India. Diabetic Medicine. 1998;15:220-7.
17. Gillman MW, Rifas-Shiman S, Berkey CS, Field AE, Colditz GA. Maternal gestational diabetes, birth weight, and adolescent obesity. Pediatrics. 2003;111:e221-6.

For peer review only - http://bmjopen.bmj.com/site/about/guidelines.xhtml

18. Sobngwi E, Boudou P, Mauvais-Jarvis F, Leblanc H, Velho G, Vexiau P, et al. Effect of a diabetic environment in utero on predisposition to type 2 diabetes. The Lancet. 2003;361:1861-5.
19. Yadav S, Arokiasamy P. Understanding epidemiological transition in India. Global Health Action. 2014;7(SUPP.1):1--14.
20. Brisbois TD, Farmer AP, McCargar LJ. Early markers of adult obesity: a review. obesity reviews. 2012;13(4):347-67.
21. Targets WWGN. 2025: Low birth weight policy brief. Geneva: WHO. 2014.
22. Vir SC. Improving women's nutrition imperative for rapid reduction of childhood stunting in South Asia: coupling of nutrition specific interventions with nutrition sensitive measures essential. Maternal & child nutrition. 2016;12:72-90.
23. Feldman PJ, Dunkel-Schetter C, Sandman CA, Wadhwa PD. Maternal social support predicts birth weight and fetal growth in human pregnancy. Psychosomatic medicine.62:715-25.
24. Diego MA, Jones NA, Field T, Hernandez-Reif M, Schanberg S, Kuhn C, et al. Maternal Psychological Distress, Prenatal Cortisol, and Fetal Weight. Psychosomatic Medicine. 2006;68:747-53.
25. Fekadu Dadi A, Miller ER, Mwanri L. Antenatal depression and its association with adverse birth outcomes in low and middle-income countries: a systematic review and meta-analysis. PloS one. 2020;15(1):e0227323.
26. Evans GW, Kim P, Ting AH, Tesher HB, Shannis DJDp. Cumulative risk, maternal responsiveness, and allostatic load among young adolescents. 2007;43(2):341.
27. Stewart RCJM, nutrition c. Maternal depression and infant growth–a review of recent evidence. 2007;3(2):94-107.
28. Babu GR, Garadi L, Murthy GVS, Kinra S. Effect of hyperglycaemia in pregnancy on adiposity in their infants in India : a protocol of a multicentre cohort study. 2014:1-8.
29. Babu GR, Murthy GVS, Deepa R, Kumar HK, Karthik M. Maternal antecedents of adiposity and studying the transgenerational role of hyperglycemia and insulin (MAASTHI): a prospective cohort study Protocol of birth cohort at Bangalore , India. BMC pregnancy and childbirth. 2016:1-9.
30. Anand SS, Vasudevan A, Gupta M, Morrison K, Kurpad A, Teo KK, et al. Rationale and design of South Asian Birth Cohort (START): a Canada-India collaborative study. BMC Public Health. 2013;13:79.
31. Organization WH. Diagnostic criteria and classification of hyperglycaemia first detected in pregnancy: A World Health Organization Guideline. Diabetes research and clinical practice. 2014;103:341-63.
32. Babu GR, Karthik M, Ravi D, Ana Y, Shriyan P, Hasige KK, et al. What makes the pregnant women revisit public hospitals for research? Participant engagement and retention trial in a public hospital (PERTH): an RCT protocol. BMC pregnancy and childbirth. 2018;18(1):369.
33. Lobo E, Vivekananda P, Deepa R, Babu GR. Development, design, and experience related to Android application-based data collection for the MAASTHI cohort study in India. RGUHS National Journal of Public Health. 2020;3(1):3 - 11.
34. Fernandes MC, Srinivasan K, Stein AL, Menezes G, Sumithra R, Ramchandani PG. Assessing prenatal depression in the rural developing world: a comparison of two screening measures. Archives of women's mental health. 2011;14(3):209-16.

For peer review only - http://bmjopen.bmj.com/site/about/guidelines.xhtml

35. Anand SS, Vasudevan A, Gupta M, Morrison K, Kurpad A, Teo KK, et al. Rationale and design of South Asian birth cohort (START): a Canada-India collaborative study. BMC public health. 2013;13(1):1-13.

36. Kumar VS, Jeyaseelan L, Sebastian T, Regi A, Mathew J, Jose R. New birth weight reference standards customised to birth order and sex of babies from South India. BMC pregnancy and childbirth. 2013;13:1.

37. Organization WH. ICD 10 international statistical classification of diseases and related health problems tenth revision, 2. Geneva: WHO. 2004.

38. Organization WH. Haemoglobin concentrations for the diagnosis of anaemia and assessment of severity. Vitamin and

Mineral Nutrition Information System. 2011; (WHO/NMH/NHD/MNM/11.1).

39. Program NHBPE. Classification of Blood Pressure-The Seventh Report of the Joint National Committee on Prevention, Detection, Evaluation, and Treatment of High Blood Pressure. US Department of Health And Human Services, National Institutes of Health, National Heart, Lung, and Blood Institute. 2004.

40. Misra A, Chowbey P, Makkar B, Vikram N, Wasir J, Chadha D, et al. Consensus statement for diagnosis of obesity, abdominal obesity and the metabolic syndrome for Asian Indians and recommendations for physical activity, medical and surgical management. Japi. 2009;57(2):163-70.

41. Singh T, Sharma S, Nagesh S. Socio-economic status scales updated for 2017. Int J Res Med Sci. 2017;5(7):3264-7.

42. Catalano PM. Obesity and pregnancy—the propagation of a viscous cycle? : Oxford University Press; 2003.

43. Ministry of Health and Family Welfare GoI. Select Health Parameters : A Comparative Analysis across the National Sample Survey Organization (NSSO) 42 , 52 and 60 Rounds. Ministry of Health and Family Welfare, India. 2007.

44. Seshiah V, Balaji V, Balaji MS, Sanjeevi CB, Green A. Gestational diabetes mellitus in India. The Journal of the Association of Physicians of India. 2004;52:707-11.

45. Wahi P, Dogra V, Jandial K, Bhagat R, Gupta R, Gupta S, et al. Prevalence of gestational diabetes mellitus (GDM) and its outcomes in Jammu region. The Journal of the Association of Physicians of India. 2011;59:227-30.

46. Anzaku AS, Musa J. Prevalence and associated risk factors for gestational diabetes in Jos, North-central, Nigeria. Archives of Gynecology and Obstetrics. 2013;287:859-63.

47. Ewenighi Chinwe O. 1* NHU, Dimkpa Uche3, Onyeanusi Joel C.1, Nnatuanya Isaac N. 4, Onoh Linus U. M.5, Ezeugwu Uchechukwu 6 , Onoh Gladys O.7, Ibeagi Ogubuike O.8 and Adejumo Babatunde I.9. Prevalence Of Gestational Diabetes Mellitus; Risk Factors Among Pregnant Women

(In Abakaliki Metropolis, Ebonyi State Nigeria.)

-. National Journal of Integrated Research in Medicine. 2013;4:56-61.

48. Ogurtsova K, da Rocha Fernandes J, Huang Y, Linnenkamp U, Guariguata L, Cho N, et al. IDF Diabetes Atlas: Global estimates for the prevalence of diabetes for 2015 and 2040. Diabetes research and clinical practice. 2017;128:40-50.

49. Shriyan P, Babu GR, Ravi D, Ana Y, van Schayck OC, Thankachan P, et al. Ambient and Indoor Air Pollution in Pregnancy and the risk of Low birth weight and Ensuing Effects in

For peer review only

For peer review only - http://bmjopen.bmj.com/site/about/guidelines.xhtml

522 Infants (APPLE): A cohort study in Bangalore, South India. Wellcome Open Research.
523 2020;3:133-.

524 **Figure Legend**

525 **Figure 1: Study recruitment and follow-ups in the MAASTHI cohort in India, 2016-2019**

526 The flow chart shows the recruitment to follow-up of participants into the MAASTHI cohort and the

527 reasons for drop-out

For peer review only

For peer review only - http://bmjopen.bmj.com/site/about/guidelines.xhtml

528

For peer review only

For peer review only - http://bmjopen.bmj.com/site/about/guidelines.xhtml

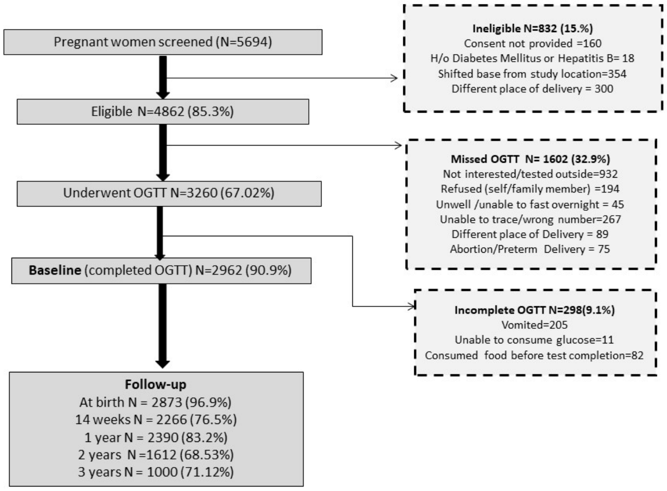

Figure 1

338x190mm (96 x 96 DPI)
